# Phosphorylation-dependent routing of RLP44 towards brassinosteroid or phytosulfokine signalling

**DOI:** 10.1242/jcs.259134

**Published:** 2021-10-20

**Authors:** Borja Garnelo Gómez, Eleonore Holzwart, Chaonan Shi, Rosa Lozano-Durán, Sebastian Wolf

**Affiliations:** 1 Centre for Organismal Studies Heidelberg, University of Heidelberg, INF230, 69120 Heidelberg, Germany; 2 Shanghai Center for Plant Stress Biology, CAS Center for Excellence in Molecular Plant Sciences, Chinese Academy of Sciences (CAS), Shanghai 201602 China; 3 Department of Plant Biochemistry, Centre for Plant Molecular Biology (ZMBP), Eberhard Karls University, D-72076 Tübingen, Germany

**Keywords:** Brassinosteroid, Cell wall, Endomembrane system, Phosphorylation, Trafficking

## Abstract

Plants rely on cell surface receptors to integrate developmental and environmental cues into behaviour adapted to the conditions. The largest group of these receptors, leucine-rich repeat receptor-like kinases, form a complex interaction network that is modulated and extended by receptor-like proteins. This raises the question of how specific outputs can be generated when receptor proteins are engaged in a plethora of promiscuous interactions. RECEPTOR-LIKE PROTEIN 44 (RLP44) acts to promote both brassinosteroid and phytosulfokine signalling, which orchestrate diverse cellular responses. However, it is unclear how these activities are coordinated. Here, we show that RLP44 is phosphorylated in its highly conserved cytosolic tail and that this post-translational modification governs its subcellular localization. Whereas phosphorylation is essential for brassinosteroid-associated functions of RLP44, its role in phytosulfokine signalling is not affected by phospho-status. Detailed mutational analysis suggests that phospho-charge, rather than modification of individual amino acids determines routing of RLP44 to its target receptor complexes, providing a framework to understand how a common component of different receptor complexes can get specifically engaged in a particular signalling pathway.

## Abstract (precis)

**Summary:** Phosphorylation of *Arabidopsis* RLP44 determines its subcellular localization and shifts the balance between its roles in promoting brassinosteroid and phytosulfokine signalling, respectively.

## INTRODUCTION

To integrate environmental cues with intrinsic developmental programs, plants depend on the perception of extracellular signals by their expanded family of cell surface receptors. The largest group of these plasma membrane-localized proteins is formed by leucine-rich repeat receptor-like kinases (LRR-RLKs), characterized by the presence of a signal peptide for entry into the secretory pathway, a leucine-rich repeat-containing extracellular domain, a single-pass transmembrane domain, and an intracellular kinase domain with homology to animal Irak/Pelle proteins (Shiu and Bleecker, 2001). LRR-RLKs form an extensive interaction network reflecting the need to decipher complex information about the environment (Smakowska-Luzan et al., 2018). LRR-RLPs, which resemble LRR-RLKs, but lack a kinase domain, contribute to RLK signalling in a variety of ways (Jamieson et al., 2018), further expanding the complexity of the network. Thus, the multitude of potential interactions and extensive sharing of components between pathways, raise a central question in plant signal transduction: how can distinct signalling responses be achieved? A partial answer is provided by the discovery of membrane sub-compartmentalization, which helps to spatially separate potential interaction partners (Galindo-Trigo et al., 2020; Gronnier et al., 2019; Jaillais and Ott, 2020). Furthermore, selective endocytosis, recycling, and eventually, the degradation of RLKs in the vacuole (Liu et al., 2020; Martins et al., 2015; Mbengue et al., 2016; Zhou et al., 2018) are crucial for tuning of signalling. Most, if not all plasma membrane receptors described so far undergo endocytosis in vesicles coated by clathrin, which depend on cytosolic adaptor complexes for cargo selection and transport (Di Rubbo et al., 2013; Gadeyne et al., 2014; Liu et al., 2020; Van Damme et al., 2011). In some, but not all, cases, ligand binding promotes internalization, possibly to establish a refractory phase after ligand exposure and prevent potentially harmful continuous activation (Nimchuk et al., 2011; Ortiz-Morea et al., 2016; Robatzek et al., 2006). Post-translational modification by phosphorylation is at the core of RLK signalling regulation. Binding of extracellular ligands to the ectodomains of several LRR-RLKs has been shown to mediate hetero-dimerization with a shape-complementary co-receptor of the SOMATIC-EMBRYOGENESIS RECEPTOR KINASE (SERK) family (Albert et al., 2013; Couto and Zipfel, 2016; Hohmann et al., 2017; Mithoe and Menke, 2018), juxtaposing their kinase domains in the cytosol. This, in turn, leads to auto and trans-phosphorylation of the kinases, resulting in an activated receptor complex capable of recruiting and phosphorylating downstream signalling components. Recent evidence suggests differential requirements for individual phosphorylation events in SERK kinase domains depending on the interacting RLK (Perraki et al., 2018). Signalling output is further modified by the activity of phosphatases and the phosphorylation-dependent release of inhibitory factors (Couto et al., 2016; Jaillais et al., 2011b; Lin et al., 2013; Monaghan et al., 2014; Park et al., 2008). In summary, post-translational modification through phosphorylation is a key mechanism to spatially and temporally control LRR-RLK-mediated signalling. One of the best-characterized LRR-RLKs is the receptor for brassinosteroid (BR) phytohormones, BRASSINOSTEROID INSENSITIVE 1 (BRI1) (Li and Chory, 1997). Signalling mediated by BRI1 and its co-receptors, such as SOMATIC-EMBRYOGENESIS RECEPTOR KINASE 1/BRI1-ASSOCIATED KINASE1 (SERK3; also known as BAK1) (Li et al., 2002; Nam and Li, 2002), plays a crucial role in cell elongation, in part by controlling a plethora of cell wall biosynthesis and remodelling genes (Belkhadir and Jaillais, 2015; Singh and Savaldi-Goldstein, 2015; Sun et al., 2010). We previously revealed that when cell wall integrity is challenged by interference with the activity of the important cell wall modification enzyme pectin methylesterase (PME), BR signalling is activated as a compensatory mechanism (Wolf et al., 2012b). This BR-mediated compensatory response depends on RECEPTOR-LIKE PROTEIN 44 (RLP44) (Wolf et al., 2014). RLP44 directly interacts with both BRI1 and BAK1 and promotes their association (Holzwart et al., 2018). However, RLP44 also interacts with and promotes the activity of the receptor complex for the plant growth peptide phytosulfokine (PSK) (Holzwart et al., 2018, 2020; Sauter, 2015). The interaction between RLP44 and the PSK receptor PSKR1 is important for the maintenance of procambial cell fate, as both RLP44 and PSK-related mutants show ectopic xylem formation in the position of the procambium in seedling roots (Holzwart et al., 2018, 2020). Thus, RLP44 acts in at least two different LRR-RLK pathways through direct interaction with their receptors. Multi-faceted interactions among LRR proteins is an emerging theme in plant receptor biology (Ma et al., 2016; Smakowska-Luzan et al., 2018); however, it is not clear how distinct responses are ensured. As RLP44 acts in two separate pathways with well-defined read-outs, it provides an excellent model to decipher how pathway specificity is achieved. Here, we show that RLP44 is phosphorylated in its highly conserved C-terminal cytosolic tail. This post-translational modification is crucial for regulating the function of RLP44 in BR signalling activation, whereas its role in PSK signalling is not affected by its phospho-status. Thus, our results provide a framework to understand how specificity can be determined in plasma membrane receptor complex interactions.

## RESULTS

### Four conserved putative phosphorylation sites are required for RLP44-mediated BR signalling activation

**Fig. 1.**
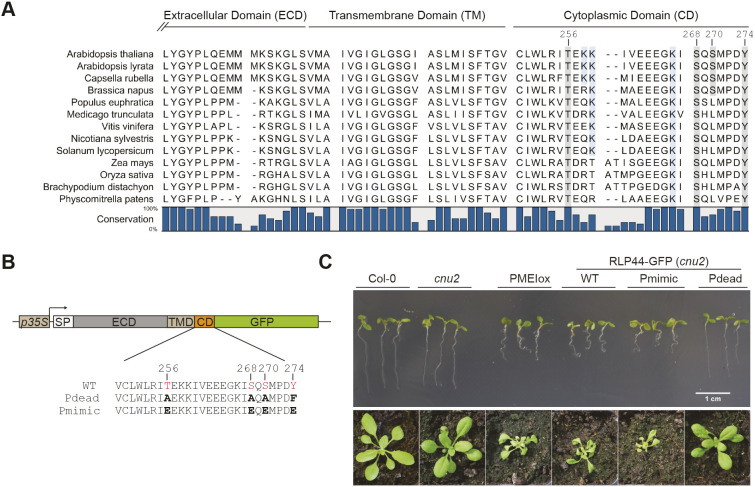
**RLP44 has an unusual C-terminal tail with four putative phosphorylation sites that are required for function.** (A) The short cytoplasmic tail of AtRLP44 contains four putative phosphorylation sites, three of which are conserved in RLP44 orthologues. The C-terminal part of the extracellular domain, predicted transmembrane domain (http://www.cbs.dtu.dk/services/TMHMM/) and cytoplasmic domain of AtRLP44 are indicated; see Fig. S1 for full alignment. (B) Schematic representation of RLP44 WT, Pdead and Pmimic variants used in this study. (C) Blocking post-translational modification of the four putative phosphosites in RLP44–GFP Pdead precludes function in the BR signalling-dependent response to cell wall modification, whereas simulating phosphorylation in RLP44–GFP Pmimic results in RLP44–GFP WT-like functionality. Expression of RLP44–GFP WT and Pmimic, but not of Pdead, is able to complement the PMEIox suppressor mutant *cnu2* and leads to recovery of the PMEIox root waving phenotype in seedlings and contorted leaf arrangement in adult plants. Images representative of three experiments, please also see Fig. S3A for additional independent transgenic lines. Image width of lower panels=4 cm.

RLP44 is unusual compared to other RLPs in *Arabidopsis thaliana*, as its juxtamembrane domain is not acidic, and its cytoplasmic C-terminal tail shows a pI of 4.7, whereas the majority of *Arabidopsis* RLPs harbour cytoplasmic tails with a basic pI (Gust and Felix, 2014). However, this unusual cytoplasmic domain (CD) is well conserved among the apparent RLP44 orthologues in land plants (Fig. 1A; Fig. S1). Interestingly, of four putative phosphorylation sites in *Arabidopsis* RLP44 (from hereon RLP44), three – T256, S268 and Y274 – are conserved in all orthologues, whereas S270 seems to be specific to Brassicacea. All four sites are predicted to be phosphorylated (NetPhos 3.1 server; www.cbs.dtu.dk/services/NetPhos/) and we have previously obtained evidence for serine phosphorylation in RLP44 using anti-phosphoserine antibodies (Wolf et al., 2014). To assess which of the four amino acids are phosphorylated *in vivo*, we performed mass spectroscopy after immunoprecipitation from transgenic *Arabidopsis* plants expressing an RLP44–GFP fusion protein under the control of the CaMV35S promoter, as well as from transiently transformed *Nicotiana benthamiana* leaves. Peptide coverage was quite poor, in particular from *Arabidopsis*, despite effective immune-purification of the RLP44 fusion protein. However, we were able to identify S268 phosphorylation in *N. benthamiana* (Fig. S2). As we could not rule out modification of the other three residues, we first assessed the effect of blocking post-translational modification of all four putative phospho-sites. To this end, we generated a version of RLP44 fused to GFP in which all four sites are mutated to either alanine (T256A, S268A, S270A) or phenylalanine (Y274F) and termed this ‘phospho-dead’ variant RLP44–GFP Pdead. Conversely, we created a ‘phospho-mimic’ (Pmimic) version of RLP44–GFP in which all four putative phospho-sites are converted to glutamate (T256E, S286E, S270E, Y274E). Throughout the article, we refer to these genetic modifications of putative phosphorylation sites as affecting ‘phospho-status’ for brevity. Constructs encoding these mutant versions of RLP44–GFP along with a wild-type version (RLP44–GFP WT) were used to transform the *rlp44* loss-of-function mutant *cnu2* (Wolf et al., 2014) for complementation assays. We originally described *cnu2* as a suppressor mutant of an overexpression line of *PMEI5* (PMEIox), which displays growth defects due to a compensatory boosting of brassinosteroid signalling strength. Activation of BR signalling in response to PMEI-mediated reduction of PME activity critically depends on RLP44, which directly interacts with the BR receptor BRI1 and its co-receptor BAK1. Mutation of RLP44 in *cnu2* thus leads to relatively normal growth despite the presence of the PMEIox transgene. As previously described (Wolf et al., 2014), expression of RLP44–GFP is able to complement *cnu2* and results in recovery of the PMEIox seedling root waving phenotype in several independent transgenic lines (Fig. 1C; Fig. S3A). Similarly, the Pmimic variant of RLP44–GFP could restore or even slightly enhance the PMEIox phenotype, suggesting that the presence of the native version of the four mutated RLP44 sites is not essential for function in BR signalling activation, and that mimicking phosphorylation at these sites might be associated with enhanced activity. In contrast, the Pdead version consistently failed to complement *cnu2* with respect to the root waving phenotype (Fig. 1D; Fig. S3A). Concentrating on one line for each construct with comparable RLP44–GFP expression levels (Fig. S3B), we made similar observations for other previously described PMEIox phenotypes (Wolf et al., 2012b), such as altered expression of BR marker genes (Fig. S3C,D), reduced seed yield (Fig. S3E) and agravitropic growth on vertical agar plates in the dark due to enhanced BR signalling (Fig. S4). In each case, the line expressing the WT or Pmimic version of RLP44-GFP in the *cnu2* suppressor mutant behaved like PMEIox, whereas the line expressing the Pdead version behaved like *cnu2* (i.e. similar to WT). We then assessed the ability of the three RLP44–GFP versions to rescue *rlp44* phenotypes in the absence of PMEIox-induced cell wall challenge. Mutants of RLP44 such as *rlp44^cnu2^* show reduced petiole length (Wolf et al., 2014), presumably caused by impaired BR signalling. Expression of RLP44–GFP WT and RLP4–GFP Pmimic, but not RLP44–GFP Pdead, could restore the petiole length defect of *rlp44^cnu2^* (Fig. S5), in line with the assumption that this phenotype is BR signalling related. In conclusion, blocking phosphorylation of four putative phosphosites in the cytoplasmic domain precluded BR signalling activation-related functions of RLP44. In contrast, introducing a negative charge to mimic phosphorylation at these sites resulted in functionality comparable to that of the WT version of RLP44–GFP.

### Phospho-status affects the subcellular localization of RLP44–GFP

**Fig. 2.**
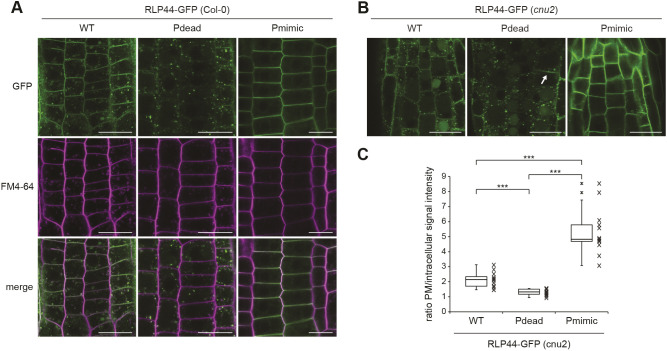
**RLP44–GFP phospho-status determines its subcellular localization.** (A) RLP44–GFP WT-derived fluorescence is observed at the plasma membrane and in the endomembrane system, as previously demonstrated (Wolf et al., 2014). RLP44–GFP Pdead is mostly confined to endomembranes, whereas RLP44–GFP Pmimic shows prominent plasma membrane localization. Scale bars: 20 µm. (B) The subcellular distribution of RLP44–GFP variants is maintained in the *cnu2* background. Arrow points to a fusing cell plate that shows increased RLP44-GFP Pdead fluorescence. Scale bars: 50 µm. (C) Quantification of mean plasma membrane to intracellular fluorescence ratio. Boxes indicate range from 25th to 75th percentile, horizontal line indicates the median, whiskers indicate data points within 1.5 times the interquartile range. Markers above whiskers indicate outliers, markers alongside box and whisker diagrams indicate individual data points, *n*=12 measurements (cells) in three independent roots for each genotype. ***P<0.001 (unpaired two-tailed Student's *t*-test).

**Fig. 3.**
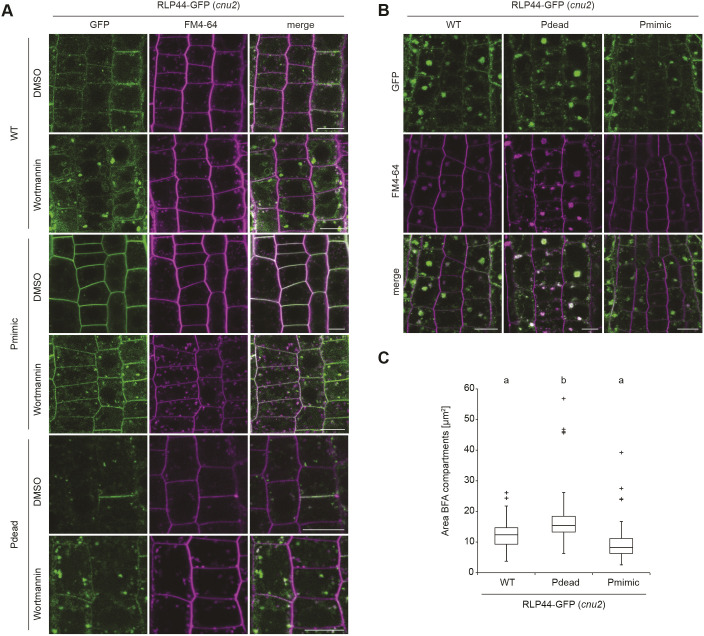
**RLP44–GFP variants undergo endocytosis.** (A) Fluorescence derived from RLP44–GFP WT, Pdead and Pmimic variants accumulates in enlarged structures after 20 µM wortmannin (Wm) treatment for 165 min, suggesting they reach late endosomes. Note increased plasma membrane labelling of RLP44–GFP Pdead after Wm treatment. Scale bars: 10 µm. (B) Fluorescence derived from RLP44–GFP WT, Pdead and Pmimic variants accumulates in BFA bodies. Roots were treated with 50 µM of BFA or DMSO for 120 min and with FM4-64 for 20 min before imaging. Scale bars: 10 µm. (C) Image quantification reveals largest fluorescent area in BFA bodies of RLP44–GFP-derived fluorescence (lower panel), *n*=116 (WT), *n*=102 (pdead), *n*=90 (Pmimic) measurements in 18 independent roots for each genotype. Boxes indicate range from 25th to 75th percentile, horizontal line indicates the median, whiskers indicate data points within 1.5 times the interquartile range. Markers above whiskers indicate outliers. Different letters indicate statistically significant differences (*P*<0.05) from other letter groups according to Mann–Whitney U-tests.

**Fig. 4.**
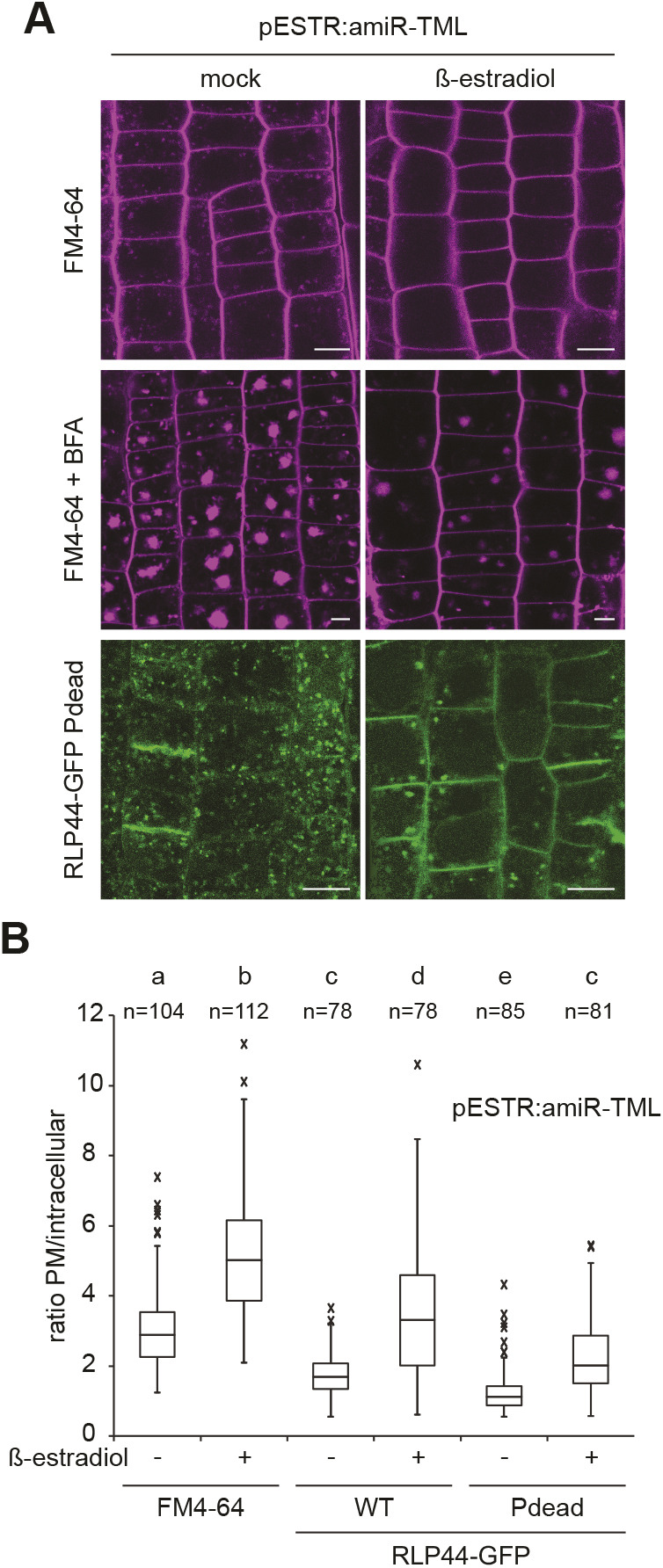
**Blocking phosphorylation promotes endocytosis of RLP44-GFP.** (A) Inducible expression of amiRNAs targeting the clathrin adapter complex TPLATE leads to reduced formation of endosomes and decreased uptake of FM4-64 into BFA bodies compared to mock control, suggesting efficient blockage of clathrin-mediated endocytosis. Knockdown of TPLATE in RLP44-GFP Pdead leads to enhanced plasma membrane localization. Scale bars: 10 µm. (B) Quantification of mean plasma membrane to intracellular fluorescence ratio in the indicated genotypes with and without amiRNA-mediated knock down of TPLATE. Boxes indicate range from 25th to 75th percentile, horizontal line indicates the median, whiskers indicate data points within 1.5 time the interquartile range. Markers above whiskers indicate outliers; *n* (cells) from 15–18 independent roots each are indicated. Different letters indicate statistically significant differences (*P*<0.05) from other letter groups according to Dunn's post hoc test after one-way Kruskal–Wallis test.

We have previously shown that RLP44–GFP is localized in intracellular vesicles and at the plasma membrane (Wolf et al., 2014), in agreement with its association with receptors for extracellular signalling ligands. To assess whether modification of the four putative phosphorylation sites influences the subcellular localization of RLP44-GFP, we imaged root tips of transgenic lines expressing RLP44–GFP WT, Pdead or Pmimic in the wild-type (Col-0) background. RLP44–GFP WT showed the expected distribution of plasma membrane and intracellular fluorescence, which partially colocalized with the styryl dye FM4-64 (Fig. 2A). Surprisingly, RLP44–GFP Pdead showed only very faint plasma membrane fluorescence and almost exclusive labelling of intracellular vesicles that appeared to largely colocalize with FM4-64 30 min after its application, suggesting endosomal localization (Fig. 2A). In sharp contrast, RLP44–GFP Pmimic showed strongly enhanced plasma membrane labelling with few intracellular vesicles (Fig. 2A). Similar results were observed in the *cnu2* background (Fig. 2B) and confirmed by quantification of the mean plasma membrane to intracellular fluorescence ratio (Fig. 2C). These results would be consistent with two different and mutually exclusive scenarios. First, the putative phosphorylation sites might be required for targeting of RLP44–GFP, thus the Pdead fluorescence distribution might reflect a failure of the receptor to be transported to the plasma membrane, whereas mimicking phosphorylation in Pmimic might enhance transport to the surface; second, differential rates of endocytic uptake might underlie the contrasting behaviour of the Pdead and Pmimic versions of RLP44–GFP. In the latter scenario, Pdead would be transported to the plasma membrane initially, but would rapidly undergo endocytosis, whereas mimicking phosphorylation would block internalization. As the second scenario is favoured by an increased Pdead fluorescence in expanding and fusing cell plates (Fig. 2C), which exhibit rearrangement of cellular trafficking towards secretion (Richter et al., 2014), we sought to test the hypothesis that the three RLP44–GFP versions differ in their rate of endocytosis. To this end, we first interfered with endosomal trafficking by applying the phosphoinositide 3-kinase inhibitor wortmannin (Wm). This treatment leads to a swelling of the multi-vesicular bodies (MVBs)/late endosomes (LEs), thus indicating the late endosomal nature of sensitive structures (Viotti et al., 2010; Wang et al., 2009). In addition, Wm can indirectly lead to an inhibition of endocytosis (Emans et al., 2002). All three RLP44–GFP versions were sensitive to Wm, as subpopulations of intracellular GFP-positive punctate showed pronounced swelling, suggesting that RLP44–GFP reaches late endosomes (Fig. 3A). Moreover, plasma membrane labelling of RLP44–GFP Pdead was increased after Wm treatment. This is consistent with the hypothesis that this RLP44 version displays low steady state abundance at the plasma membrane because it experiences increased endocytic uptake. Consequently, inhibition of this uptake, in this case by Wm, leads to an increase in abundance at the plasma membrane (Fig. 3A). To independently corroborate these results, we made use of the fungal toxin brefeldin A (BFA), which, in *Arabidopsis* roots, leads to aggregation of endosomal compartments into a hybrid organelle, the BFA compartment, in which endocytic cargo becomes trapped (Dettmer et al., 2006; Geldner et al., 2003; Grebe et al., 2003; Viotti et al., 2010). Consequently, quantification of fusion protein-derived fluorescence in BFA compartments has been used to assess endocytosis of plasma membrane receptors (Di Rubbo et al., 2013; Martins et al., 2015). After 120 min of BFA treatment, the three RLP44–GFP versions displayed differential accumulation in BFA compartments, with the Pdead version showing the strongest signal, followed by WT and Pmimic (Fig. 3B,C). These observations are consistent with differential endocytosis as the mechanistic explanation for the different subcellular distribution of RLP44–GFP WT, Pdead and Pmimic. In order to directly test the impact of retrograde trafficking on RLP44–GFP localization through genetic interference with endocytic uptake, we used a previously described line expressing, in an inducible manner, artificial microRNAs (amiRNAs) directed against the TPLATE adapter complex to block clathrin-mediated endocytosis (Gadeyne et al., 2014; Van Damme et al., 2011). As expected, induction of amiRNA expression for 48 h led to a marked increase in the ratio of plasma membrane-localized to intracellular FM4-64 fluorescence and a dramatic reduction in size and quantity of FM4-64-positive BFA compartments (Fig. 4A,B), indicating strongly decreased endocytosis. Importantly, amiRNA induction in plants expressing RLP44–GFP Pdead led to the appearance of GFP fluorescence at the plasma membrane, in sharp contrast to mock treatment (Fig. 4A,B). Quantification of plasma membrane and intracellular GFP fluorescence in RLP44–GFP WT and Pdead lines revealed that inhibition of clathrin-mediated endocytosis leads to a WT-like fluorescence distribution of RLP44-GFP Pdead (Fig. 4B), suggesting that phospho-status at least partially governs endosomal trafficking of RLP44–GFP, although reduced secretion of RLP44–GFP Pdead cannot be excluded.

### Subcellular distribution of RLP44 is modified by BRI1

**Fig. 5.**
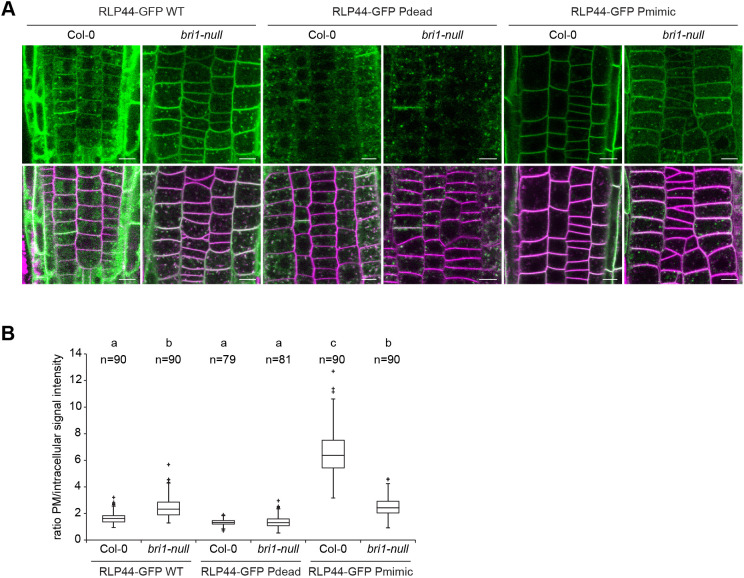
**Subcellular distribution of RLP44–GFP depends on BRI1.** (A) Comparison of fluorescence distribution derived from the three RLP44–GFP variants in Col-0 and *bri1-null* background. Note the increased appearance of intracellular fluorescence in RLP44-GFP Pmimic expressed in the *bri1-null* mutant. Scale bars: 5 µm. (B) Quantification of mean plasma membrane to intracellular fluorescence ratio in the indicated genotypes, derived from images as in A. Boxes indicate range from 25th to 75th percentile, horizontal line indicates the median, whiskers indicate data points within 1.5 time the interquartile range. Markers above whiskers indicate outliers; measurements are from 12 independent roots for each genotype with the indicated n. Different letters indicate statistically significant differences (*P*<0.05) from other letter groups according to Tukey's post hoc test after one-way ANOVA.

To assess whether the subcellular localization of RLP44–GFP can be affected by the presence of its interaction partners, we quantified the subcellular distribution of RLP44–GFP fluorescence in the absence of BRI1. To this end, we crossed RLP44–GFP WT, Pdead and Pmimic into the T-DNA insertion line *bri1-null* (Jaillais et al., 2011a), which, based on the available evidence, completely lacks BRI1 protein. Whereas neither localization of the RLP44–GFP WT version nor that of RLP44-GFP Pdead was strongly affected in the *bri1-null* mutant (Fig. 5A,B), the almost exclusive plasma membrane localization of RLP44–GFP Pmimic clearly depended on the presence of BRI1 (Fig. 5C) – intracellular labelling was strongly increased in *bri1-null* compared to the Col-0 background (Fig. 5A) and the plasma membrane-to-cytosol fluorescence ratio of Pmimic was not increased compared to the RLP44–GFP WT in the mutant background (Fig. 5B). These results suggest that, at least in part, presence of BRI1 precludes endocytosis of the Pmimic version of RLP44, although a similar effect was not observed with the WT protein. In agreement with its inability to promote BR signalling, the subcellular localization of RLP44–GFP Pdead was unaffected by the presence or absence of BRI1. However, it should be noted that the mechanistic insight provided by these data is limited by the large transcriptional re-arrangements in *bri1* loss-of-function mutants (Chaiwanon and Wang, 2015) and potential indirect effects on the composition of various receptor complexes (Holzwart et al., 2020; Smakowska-Luzan et al., 2018).

### Phosphorylation is not required for the role of RLP44 in PSK signalling

**Fig. 6.**
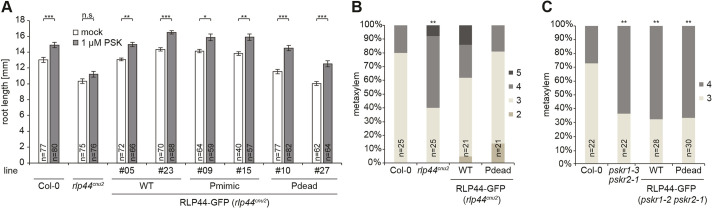
**RLP44 is functional in PSK signalling irrespective of potential phospho-status.** (A) Root length response to exogenous application of PSK peptide is impaired in *rlp44^cnu2^* (Holzwart et al., 2018), but restored by expression of all three RLP44–GFP variants, including Pdead. Bars denote mean±s.e.m. of root length after 5 days of growth on control plates or plates containing 1 µM PSK peptide. *P<0.05, **P<0.01, ***P<0.001 (Tukey's post hoc test after one-way ANOVA). (B) The PSK signalling-dependent ectopic xylem phenotype of *rlp44^cnu2^* (Holzwart et al., 2018) is restored by expression of RLP44–GFP Pdead. (C) Effect of RLP44 expression depends on the presence of PSK receptors PSKR1 and PSKR2. Bars in B and C denote frequency of roots with indicated number of metaxylem cells. **P<0.01 from Col-0 (Dunn's post hoc test with Benjamini–Hochberg correction after Kruskal–Wallis modified U-test).

We have recently described (Holzwart et al., 2018) that, apart from promoting BR signalling, RLP44 plays a role in promoting signalling mediated by the receptors of PSK peptide hormones, PSKR1 and PSKR2 (Matsubayashi et al., 2002, 2006). Having demonstrated that the role of RLP44 in BR signalling depends on the four phosphosites in the cytosolic tail, we next assessed whether the Pdead and Pmimic versions affected functionality of RLP44 in the PSK pathway. To this end, we analysed PSK-related phenotypes in complemented *rlp44^cnu2^* mutants. PSK signalling is required in the epidermis for normal root elongation (Hartmann et al., 2013; Kutschmar et al., 2009) and exogenously applied PSK peptide leads to a moderate increase in the root length of wild-type seedlings (Kutschmar et al., 2009; Ladwig et al., 2015; Wang et al., 2015). In line with its role in promoting PSK signalling, *rlp44^cnu2^* is compromised in this response (Holzwart et al., 2018, 2020). Analysis of the complementation lines described earlier (Fig. S5) revealed that all three RLP44–GFP versions, including Pdead, were able to restore the response to PSK in *rlp44^cnu2^* (Fig. 6A), suggesting that PSK and BR signalling might have different requirements for the modification of the four RLP44 phosphorylation sites. Notably, the PSK signalling-mediated promotion of root growth is believed to occur in the epidermis, a tissue where *RLP44*, *BRI1* and *PSKR1* are co-expressed (Friedrichsen et al., 2000; Kutschmar et al., 2009; Holzwart et al., 2018; Matsubayashi et al., 2006). To corroborate these results, we tested whether RLP44–GFP Pdead could complement the ectopic xylem phenotype of *rlp44^cnu2^* seedling roots, which is caused by reduced PSK signalling (Holzwart et al., 2018). In line with the root length assay, expression of RLP44–GFP Pdead led to wild-type-like xylem cell numbers (Fig. 6B), confirming that phosphorylation of these residues is not a requirement for the role of RLP44 in promoting PSK signalling. To exclude a PSK signalling-independent effect of RLP44 Pdead on xylem cell numbers we performed a rescue experiment in *pskr1-3 pskr2-1* double mutants. As previously described for the WT RLP44–GFP (Holzwart et al., 2018), RLP44 Pdead requires the presence of PSK receptors to exert any effect on xylem cell number (Fig. 6C). In summary, our results suggest that phosphorylation modulates RLP44 functionality in different receptor complexes.

### Phospho-charge, rather than modification of individual amino acids determines RLP44 functionality in BR signalling

**Fig. 7.**
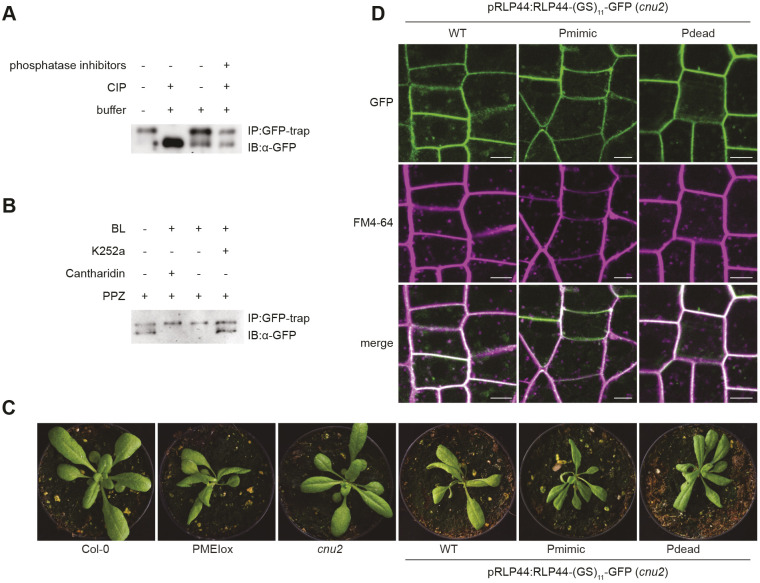
**Overall charge, rather than specific phosphosites modulate RLP44 function.** (A) Phosphatase treatment of pRLP44:RLP44-(GS)_11_-GFP leads to a pronounced shift in electrophoretic mobility, visualized by western blotting using GFP antiserum. (B) The relative abundance of the lower mobility, phosphatase-sensitive RLP44–(GS)11–GFP band is responsive to 24-Epibrassinolide (BL) and kinase inhibitor treatment. (C) The Pdead version of pRLP44:RLP44-(GS)11-GFP is able to complement *cnu2* in a similar manner to the WT and Pmimic versions. (D) All three RLP44–(GS)_11_–GFP versions mainly accumulate at the plasma membrane. Scale bars: 5 µm. Images representative of three experiments. Image width=7.25 cm.

During the course of this work, we noticed that a previously characterized RLP44 line (Holzwart et al., 2018, 2020) in which a serine-rich linker [(GS)_11_] separates RLP44 and GFP, tended to show strong overexpression phenotypes despite being under control of the native RLP44 promoter and localized predominantly at the plasma membrane (Fig. S6A,B). To test whether serine residues in this linker might become phosphorylated, we performed phosphatase assays on immunoprecipitated RLP44 protein fusions. In contrast to RLP44–GFP (Fig. S3B), RLP44–(GS)_11_–GFP, harbouring the serine-rich linker, clearly showed an additional band of lower mobility, which disappeared after calf intestinal phosphatase (CIP) treatment, indicating that the upper band represents a phosphorylated form of the fusion protein (Fig. 7A). These results suggest that the immediate environment of the RLP44 C-terminus can undergo phosphorylation and that hyperphosphorylation in the linker might confer a gain-of-function phenotype with respect to BR signalling. Moreover, a hyperphosphorylated linker can be exploited to visualize regulation of phosphorylation. Western blotting after immunoprecipitation revealed that the relative abundance of the lower mobility (i.e. phosphorylated) form of RLP44–(GS)_11_–GFP seemed to be reduced by treatment with the kinase inhibitor K252a and increased by treatment with brassinolide (Fig. 7B). To further elucidate how hyperphosphorylation of RLP44 affects activity, we created Pdead and Pmimic versions of RLP44–GFP harbouring the serine-rich linker. In contrast to what was observed with the previous RLP44–GFP constructs, all three RLP44–(GS)_11_–GFP versions complemented the *cnu2* rosette phenotype and showed almost exclusive plasma membrane localization (Fig. 7C,D). Moreover, the two RLP44 mutant versions showed a slower-migrating form similar to the phosphorylated form of RLP44–(GS)_11_–GFP, consistent with phosphorylation of the linker independently of the amino acid composition of the RLP44 cytosolic domain (Fig. S6C). This suggests that hyperphosphorylation increases RLP44 activity with respect to BR signalling and shifts its localization to the plasma membrane. In addition, these results would suggest that overall charge, rather than modification of specific amino acids, is the determining factor in controlling RLP44 activity.

**Fig. 8.**
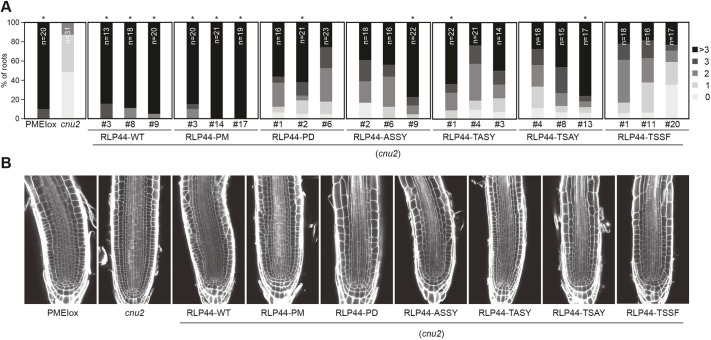
**RLP44 phosphosites are crucial for its function in the native protein context.** (A) Mutation of individual putative phosphosites in untagged RLP44 constructs results in reduced restoration of the PMEIox cell wall orientation phenotype in the *cnu2* background. Upper panels show quantification of aberrant cell wall orientation in median sections of root meristems. Asterisks indicate statistically significant difference (*P*<0.05) from *cnu2* according to Kruskal–Wallis non-parametric test followed by Chi-square analysis after Bonferroni adjustment. (B) Representative root meristem CLSM images of the indicated genotypes after staining with Calcofluor White and ClearSee (Kurihara et al., 2015) treatment. Images representative of three experiments.

It is not surprising that attaching a C-terminal fusion partner to a receptor protein might alter its activity in different ways. Therefore, the three 35S-driven RLP44–GFP versions might accentuate phosphorylation-mediated tuning of RLP44 activity at the plasma membrane. To elucidate the role of RLP44 phosphorylation independent of the linker sequence, we generated constructs encoding untagged RLP44 with single mutations in individual phosphosites as well as quadruple Pmimic and Pdead versions. We used these constructs along with the WT RLP44 version to complement *cnu2* (Wolf et al., 2014) and scored the aberrant cellular morphology phenotype described for PMEIox (Li et al., 2021 preprint), as this represents the most sensitive read-out of RLP44 function. As expected, we found that the RLP44 WT construct led to a reversion of the *cnu2* phenotype, resulting in root meristems that were indistinguishable from PMEIox (Fig. 8). In contrast, RLP44 versions in which individual phosphosites were mutated to alanine (T256A, S268A, S270A) or phenylalanine (Y274F) resulted in strongly reduced reversion of the *cnu2* phenotype (Fig. 8). A similar phenotype was displayed by the Pdead version, while Pmimic fully complemented *cnu2*. Even though these experiments suggest a more nuanced effect of the phosphosites in untagged RLP44, they broadly confirm our previous results. It should also be noted that, based on quantitative real-time RT-PCR (qRT-PCR) analysis, all untagged complementation lines show rather high expression of the RLP44 transgene, despite being driven from the *RLP44* promoter (Fig. S6D). There are several conceivable explanations for this, ranging from an incomplete, hyperactive promoter fragment to increased mRNA stability in the transgene due to the recombinant 3′ environment. Nevertheless, these results are consistent with the notion that RLP44 function is modified by the phospho-state of its C-terminus. Taken together, our results show that availability of RLP44 to engage with the BRI1 or PSKR1 receptor complexes is differentially modulated by phosphorylation, and demonstrates how selectivity and specificity of different receptor-mediated signalling pathways can be encoded in the pattern of post-translational modifications.

## DISCUSSION

Plants rely on a complex network of cell surface receptors to integrate developmental and environmental cues into behaviour adapted to the conditions. In light of the many possible interactions between LRR-RLKs (Smakowska-Luzan et al., 2018) and the high likelihood of accidental encounters in the crowded plasma membrane, which restricts mobility to two dimensions, a central question in signal transduction is thus how a specific response that transcends noise is ensured. In the current model of LRR-RLK signalling activation, the extracellular ligands serve as molecular tether to bring receptor and co-receptor together and thereby juxtapose their kinase domains in the cytosol, so that trans-phosphorylation can ensue (Hohmann et al., 2017). Fully activated kinase domains then recruit downstream signal transduction components, which can be dedicated to individual signalling pathways (Brutus et al., 2010; He et al., 2000; Hohmann et al., 2018). For example, a chimeric receptor constituted by the BRI1 extracellular domain and the cytosolic domain of the immune receptor XA21 triggers immune signalling in response to BRs (He et al., 2000), whereas a similar BRI1-HAESA chimera is able to complement *haesa* mutants in a BR-dependent manner (Hohmann et al., 2018). However, several pathways share components, as demonstrated by the ubiquity of SERK co-receptors (Ma et al., 2016), which are also an essential component of both PSK and BR signalling (Ladwig et al., 2015; Wang et al., 2015), the two LRR-RLK-governed pathways studied here. In addition to RLKs, RLPs contribute to receptor-mediated signalling and increase the complexity of the plasma membrane receptor network. We have previously demonstrated that RLP44 can promote both PSKR1- and BRI1-mediated signalling, but it was unclear how these activities are coordinated in light of the fact that RLP44 seems to act independently of the BR and PSK ligands (Holzwart et al., 2018; Wolf et al., 2014). Here, we show that phospho-status can route RLP44s towards functioning in PSK or BR signalling. This is consistent with previous proteomic analysis that revealed that putative phosphorylation sites are enriched in the binding interfaces of heterooligomers (Nishi et al., 2011) and proteins that engage in multiple (mutually exclusive) interactions using the same contact surface, presumably acting as a switch between different pathways (Tyagi et al., 2009). While we could only demonstrate phosphorylation of S268 in plants, modification of T256, S270 and Y274 by a negative charge generally seems to promote function in the BRI1-mediated signalling pathway.

Post-translational modifications play a central role in regulating the trafficking of plasma membrane proteins. It is well established that ubiquitylation can act as a signal for internalization and endosomal sorting across kingdoms (Dubeaux and Vert, 2017; Dubeaux et al., 2015; Haglund and Dikic, 2012). Interestingly, RLP44 shows a highly conserved lysine at position 266. It is thus tempting to speculate that phosphorylation might affect ubiquitylation (Swaney et al., 2013; Venne et al., 2014), as previously observed with plasma membrane proteins such as EGFR (Sigismund et al., 2013). However, the effect of phospho status on RLP44 membrane localization seems to occur independently of ubiquitin since, in our mass spectrometry data, we found the characteristic ubiquitin di-glycine remnant (after trypsin digestion) on K266 of both RLP44–GFP WT and RLP44–GFP Pdead. Although these data do not rule out an impact of phosphorylation on the type of ubiquitylation, they do show that phosphorylation is not required for the decoration with ubiquitin per se. Increasing the charge of the RLP44 C-terminus could promote plasma membrane localization through different means, favouring the interaction with BRI1. However, our results point to a slightly different scenario – RLP44 might be constitutively primed for endocytosis, and its dwell time at the membrane might be determined by the interactions it engages in. In favour of this hypothesis, endosomal uptake of RLP44–GFP Pmimic was strongly enhanced in the absence of BRI1, although indirect effects, for example, transcriptional rearrangements, cannot be excluded. Notably, the RLP44–GFP phospho-status had no impact on its functionality in the PSK pathway, despite RLP44–GFP Pdead showing very low abundance at the plasma membrane. This raises the question of whether PSKR1 signalling can occur from endosomes, as it is assumed for diverse signalling pathways in animals (Howe and Mobley, 2004; Sigismund et al., 2012) and also discussed in plants (Geldner and Jurgens, 2006). Alternatively, a brief presence of RLP44–GFP Pdead at the plasma membrane might be sufficient for supporting PSK signalling. To assess whether membrane sub-compartmentalization contributes to the maintenance of specificity, it will be important to track trafficking of RLP44 together with its interaction partners BRI1 and PSKR1 using advanced imaging technology (Bucherl et al., 2017; McKenna et al., 2019).

We found apparent orthologues of RLP44 in all analysed plant species, suggesting that RLP44-like genes are under strong selective pressure, similar to other RLPs involved in development such as CLV2 and TMM (Fritz-Laylin et al., 2005). Notably, T256, S268 and Y274 are strictly conserved in all analysed RLP44 orthologues, despite the tendency of phosphorylation sites to diverge rapidly in linear motifs and receptor proteins (Beltrao et al., 2012; Holt et al., 2009; Riaño-Pachón et al., 2010). In contrast, S270 is only found in *Brassicacea* and does not substitute an acidic residue, which is assumed to contribute a dynamic switch to a pre-existing interaction (Pearlman et al., 2011). In this respect, it is noteworthy that we identified RLP44 as required for the response to changes in pectin de-methylesterification in *Arabidopsis* (Wolf et al., 2014). This cell wall modification apparently evolved from an ancient cell wall consolidation mechanism and is operative in extant members of the charophytes (Domozych et al., 2014; Nishiyama et al., 2018; Popper et al., 2011; Proseus and Boyer, 2006; Wolf et al., 2012a). On the other hand, BRI1-like brassinosteroid receptors and PSK perception seem to be restricted to seed plants (Cheon et al., 2013; Bowman et al., 2017), thus RLP44 orthologues could predate some of its interaction partners. It will be interesting to dissect how a protein like RLP44, which modulates the function of distinct RLKs, co-evolved with its interaction partners.

## MATERIALS AND METHODS

All experiments have been repeated at least twice. All reagents were obtained from Sigma-Aldrich/Merck (Darmstadt, Germany) if not stated otherwise.

### Plant material and growth conditions

All plants used in this study were of the Col-0 ecotype *of Arabidopsis thaliana (L.) Heynh.* and are described in Table S1. Seeds were sterilized with 1.2% NaOCl in 70% ethanol and washed twice with absolute ethanol, and dried under sterile conditions. If not indicated otherwise, plants were grown in half-strength MS medium supplemented with 1% sucrose and 0.9% plant agar (Duchefa, Haarlem, The Netherlands). For treatment with ConcA and MG132, plants were grown for 6 days on standard medium on plates and transferred to liquid half-strength MS medium containing DMSO (mock treatment), 50 µM MG132, or 1 µM ConcA for 5 h before sample collection and immunopurification. To study the seedling growth response to PSK, plates were supplied with 10 nM α-PSK (PolyPeptide, Strasbourg, France). Roots were measured after 6 days of growth in long day conditions (8 h darkness, 16 hours light of 125 μmol m^−2^ s^−1^) using Image J (https://imagej.nih.gov/ij/). Average or average normalized to the mock control together with the standard deviations of at least 50 plants were plotted for each experiment. To study the phosphorylation status of RLP44 in different mutant backgrounds, ∼100 seeds per well were disposed in six-well-plates containing 5 ml of liquid half-strength MS medium supplied with 1% sucrose. After stratification, plates were placed into growth chambers with long day conditions and kept in agitation (100 rpm). At 3 days after germination, seedlings were treated with 5 µM propiconazole (PPZ). At 6 days after germination and 3 h before harvesting, seedlings were treated independently with 50 µM cantharidin, 2 µM K-252a (Enzo Life Sciences, Lörrach, Germany) or 50 µM of DMSO. At 1.5 h before harvesting, seedlings were treated with 1 µM 24-Epibrassinolide (BL) or 80% ethanol. Afterwards, plant material was carefully collected, weighed and directly froze in liquid N_2_.

### Cloning

RLP44–GFP under control of the CaMV 35S promoter was generated by amplifying the coding sequence of the (intronless) *RLP44* from genomic DNA using primers SW660 and SW670 (all primer sequences are given in Table S2), and subsequent Gateway cloning into pDONR207 and pK7FWG2 (Karimi et al., 2002). RLP44–GFP Pdead was generated by introducing the T254A mutation with site-directed mutagenesis using primers SW666 and SW667 and RLP44 in pDONR207 as template. After recombination reaction into pK7FWG2, this plasmid was used as a PCR template to introduce the remaining three mutations with primers SW668 and SW660. The resulting PCR product was introduced in pDNOR207 through BP reaction, after which a sequence-confirmed clone was used for LR reaction into pK7FWG2. RLP44–GFP Pmimic was created analogously using primers SW672 and SW673 to introduce the T256E mutation and primers SW671 and SW660 to introduce the remaining mutations via PCR. The resulting PCR product was introduced in pDNOR207 through BP reaction, after which a sequence-confirmed clone was used for LR reaction into pK7FWG2. The pRLP44:RLP44-(GS)_11_-GFP Pdead and Pmimic constructs were generated by using primer SW1179 and SW1367 (Pdead) or SW1368 (Pmimic) with the Pdead and Pmimic constructs described above. The resulting PCR products were ligated into pGGC000 to generate entry clones. Destination clones for plant transformation were assembled with GreenGate cloning as described in Table S3. The WT pRLP44:RLP44-(GS)_11_-GFP construct was described before (Holzwart et al., 2018). In-fusion cloning (Takara Bio Europe, Saint-Germain-en-Laye, France) with primers 2446-2455 was used to generate a second set of mutant constructs in entry vector pGGC000 (Lampropoulos et al., 2013), fusing RLP44 variants to GFP, separated by a GA linker. The GFP portion was amplified with primers SW using vector pK7FWG2 as template. RLP44 fragments were amplified using the corresponding construct described above as template as follows: WT with SW2446+SW2447, Pdead with SW2446+SW2454, Pmimic with SW2446+SW2455, ASSY with SW2446+SW2447, TASY with SW2446+SW2447, TSAY with SW2446+SW2450, and TSSF with SW2446+SW2452. GreenGate cloning was performed to generate the RLP44 version under control of its own promoter (Table S3). Untagged RLP44-WT, RLP44 Pdead, RLP44 Pmimic and RLP44 single mutations under control of the endogenous RLP44 promoter were generated by using the previously described constructs as template for PCR reactions using primer SW3000 and one of the primers SW3001-3006 (Table S2). Subsequently, Gateway cloning into pDONR207 and pGWB501 was performed.

### Immunopurification and mass spectroscopy analysis

Immunopurification from *Arabidopis* seedlings and western blotting were performed as described previously (Holzwart et al., 2018). For analysis of phosphorylation of the pRLP44:RLP44-(GS)11-GFP line, seedlings were grown in liquid culture for 4 days, harvested and proteins were extracted as previously described (Holzwart et al., 2018). Treatment with five units of calf intestinal phosphatase in the presence and absence of phosphatase inhibitors was carried out for 1 h, after which samples were boiled in SDS sample buffer and subjected to western blotting as previously described (Holzwart et al., 2018) using anti-GFP (Biolegend cat #902605, Biozol, Eching, Germany) purified mouse monoclonal IgG, diluted 1:10,000 in Tris-buffered saline plus 0.1% Tween 20 (TBST) plus 3% BSA and anti-mouse polyclonal IgG peroxidase conjugate (A9044, Sigma-Aldrich), diluted 1:10,000 TBST plus 3% BSA. For mass spectroscopy experiments, RLP44–GFP (WT or mutant variants) were transiently expressed in three- to four-week-old *N. benthamiana* leaves by agroinfiltration as previously described (Holzwart et al., 2018). Samples were taken 2 days post-infiltration; accumulation of the GFP-fused proteins was confirmed by confocal microscopy. Plant tissue was ground in liquid nitrogen, total proteins were extracted by adding lysis buffer (100 mM Tris-HCl pH 8.0, 150 mM NaCl, 10% glycerol, 5 mM EDTA, 5 mM DTT, 1 mM PMSF, 1% protease inhibitor cocktail and 1% NP-40) and the extracts were cleaned by filtration; extracts were incubated with GFP-Trap beads (#gta-20, Chromotek, Planegg-Martinsried, Germany) for 1 h, and beads were subsequently washed using washing buffer with detergent (100 mM Tris-HCl pH 8.0, 150 mM NaCl, 10% glycerol, 2 mM DTT, 1% protease inhibitor cocktail and 0.2% NP-40) three times and washing buffer without detergent (100 mM Tris-HCl pH 8.0, 150 mM NaCl, 10% glycerol, 2 mM DTT, 1% protease inhibitor cocktail) twice. Mass spectrometry analysis was performed at the Proteomics Core Facility of the Shanghai Center for Plant Stress Biology. Matching raw MS data to peptide sequences was performed using Mascot software with the annotated proteins from the *N. benthamiana* draft genome sequence v. 0.4.4, which was obtained from the International Solanaceae Genomics Project (SOL) (https://solgenomics.net/), and AtRLP44–GFP sequence.

### Quantitative real-time PCR

For RNA analysis, a maximum of 100 mg of frozen *A. thaliana* seedling material was ground in a 2 ml reaction tube with the aid of a pre-cooled tissue lyser (TissuelyserII, Qiagen, Hilden, Germany). RNA from ground tissue was extracted with an RNA purification kit (Roboklon, Berlin, Germany), following the manufacturer's instructions. Synthesis of cDNA was carried out using AMV Reverse Trascriptase (EURx, Gdansk, Poland) following the manufacturer's instructions. The cDNA reaction was diluted 1:10 in water and used for qPCR analysis with primers directed against BR marker genes *EXPA8* and *DWF4* or against *RLP44*. The SYBRR Green I nucleic acid gel stain was used for detection, *CLATHRIN* (At1g10730) was used as reference gene. qPCR reactions were run in a Rotor-Gene Q 2plex (Qiagen, Hilden, Germany) and the amplification data extracted by the 75 Rotor-Gene Q 2plex software and analysed according to the method of Muller et al. (2002). For primers, see Table S2. To assess *RLP44* transcript levels, PMEIox or *cnu2* seedlings transformed with untagged RLP44 constructs were ground in liquid nitrogen and RNA purification was performed with the OMEGA BIOTEK kit (OMEGA BIOTEK, Norcross, GA, USA) following the manufacturer's instructions. DNAase treatment and cDNA synthesis were performed with the iSript gDNA clear cDNA synthesis kit (BIO-RAD, Hercules, CA, USA). qRT-PCR was performed using *RLP44* primers listed in Table S2 and *ACTIN* (*ACT2*) as reference gene.

### Genotyping

Presence of the *rlp44^cnu2^* mutation was assessed by CAPS marker using primers SW503 and SW504 and subsequent *Hinf*I digestion. For the genotyping of the *bri1-null* T-DNA insertion, primers SW1378 and SW1379 were used for detection of the wild-type allele. Presence of the T-DNA insertion was assessed with primers SW1377 and SW1379. For genotyping *pskr1-3*, primers SW1745 and SW1746 were used to assess the presence of the wild type allele, and SW130 and SW1746 to assess the presence of the T-DNA insertion. Primers SW1984 and SW1985 were used to detect the presence of the *PSKR2* wild-type allele, and SW230 and SW1985 were used to detect the T-DNA. Presence of Gateway insertions was checked by using primers SW905 and SW906 directed against the attB1 and attB2 sequences, respectively. Presence of GreenGate insertions was assessed by PCR with primers SW1202 and SW1137. All primer sequences are given in Table S2.

### Xylem cell number analysis

Basic fuchsin staining of 5-day-old seedling roots and CLSM analysis was performed as described (Holzwart et al., 2018).

### Microscopy

Confocal laser scanning microscopy (CLSM) of *Arabidopsis* roots and *N. benthamiana* leaf discs was performed on a TCS SP5 II inverted confocal laser scanning microscope (Leica, Mannheim, Germany) or a LSM 510 Meta confocal laser scanning microscope (Zeiss, Oberkochen, Germany). In the first case, a HCX PL APO lambda blue 63.0×1.20 water immersion objective (Leica, Mannheim, Germany) was used. In the second case, Plan-Neofluoar 5.0×1.05, Plan-Neofluar 25.0×0.80 water immersion and C-Aprochromat 63.0×1.20 water immersion objectives were used. Excitation wavelength was set to 488 nm for GFP, 514 nm for YFP, and 561 nm for RFP or mCherry. Emission was recorded at 500–545 nm for GFP, at 545–573 nm for YFP and 620–670 nm for RFP or mCherry using HyD hybrid detectors (Leica, Mannheim, Germany) or photomultipliers (PMT) detectors (Zeiss, Oberkochen, Germany). For inhibitor treatments, 6-day-old seedlings were incubated in 12-well plates using half-strength liquid MS, pH 5.8 supplemented with 20 μM wortmannin (Wm) or 50 μM of brefeldin A (BFA). For mock treatment, an equivalent volume of DMSO was used. Incubation with inhibitors took place at 22°C in the dark for 165 min (Wm) or 120 min (BFA) before imaging. FM4-64 staining was performed in half-strength liquid MS, pH 5.8 with 1 μM FM4-64 (Molecular Probes/ThermoFisher Scientific, Erlangen, Germany) for 20 min. Seedlings were imaged with CLSM using 561 nm laser line for excitation and 670–750 nm range for emission detection. For quantification of oblique cell walls in root meristems, seedlings were grown on half-strength MS pates for 5 days, fixed in 4% paraformaldehyde solution, washed in PBS twice and incubated in ClearSee solution overnight (Kurihara et al., 2015; Ursache et al., 2018). Seedlings were stained for 60 min with 0.1% (w/v) Calcofluor White in ClearSee solution and subsequently washed for 30 min in ClearSee (Ursache et al., 2018). Seedlings were imaged using 405 nm laser line for excitation and 425–475 nm range for emission detection.

### Plasma membrane-intracellular signal ratio measurement

Images for were processed using Fiji (https://imagej.net/software/fiji/). Background was subtracted with a rolling ball radius of 50.0 pixels. A total of 8–10 cells per image were chosen for measurements and a fixed region of interest (ROI) covering the plasma membrane (PM) was used to measure the area, mean grey value, minimum and maximum grey value. The same parameters were measured on the corresponding intracellular region directly below the PM using the same ROI size and shape. Ratios were obtained by dividing the mean grey value of the PM ROI by the mean value of its corresponding intracellular ROI.

## Supplementary Material

Supplementary information

Reviewer comments
