# Supplementary material for: Phosphorylation-dependent routing of RLP44 towards brassinosteroid or phytosulfokine signalling
Source: J Cell Sci. 2021 Oct 20;134(20):jcs259134. doi: 10.1242/jcs.259134 (PMC8572011; doi:10.1242/jcs.259134)
Supplement: Supplementary information [file joces-134-259134-s1.pdf]

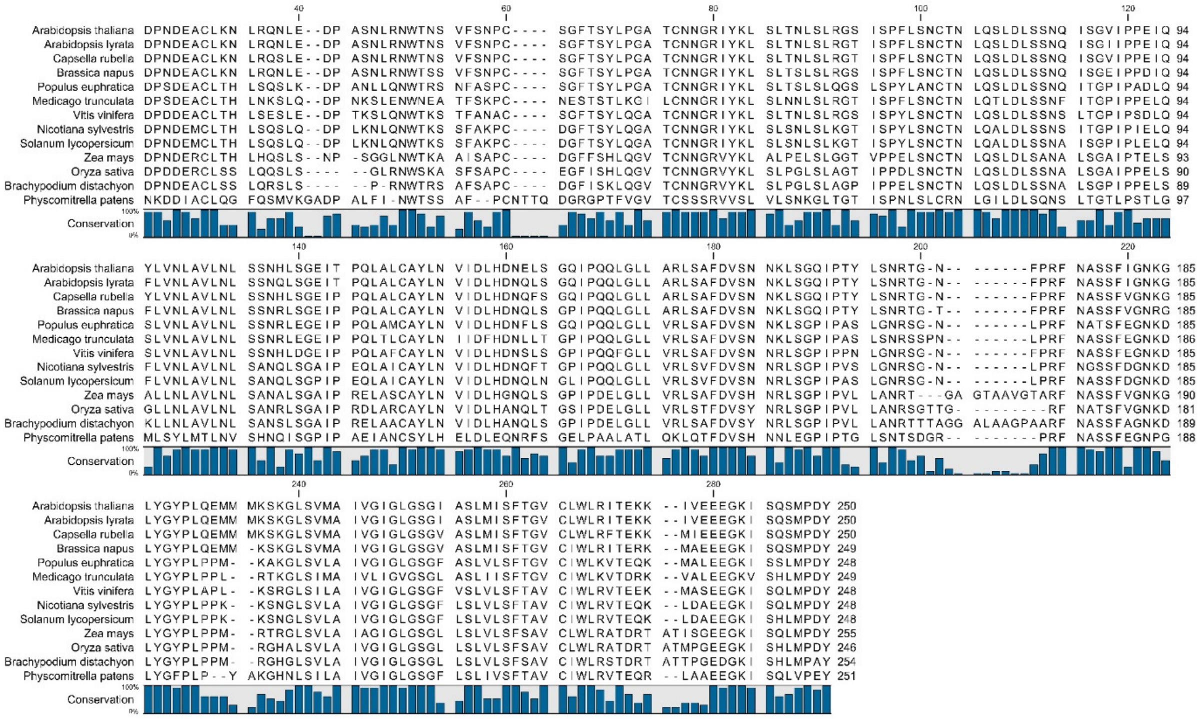

Fig. S1. Alignment of RLP44 amino acid sequences from various plant species.

Journal of Cell Science • Supplementary information

A

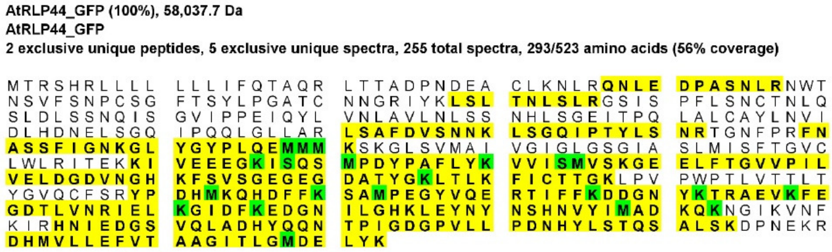

B

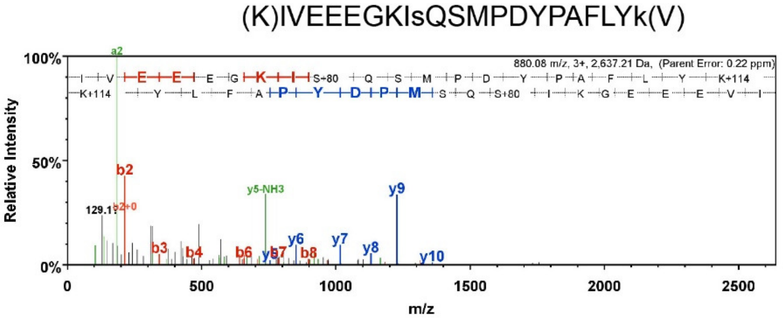

C

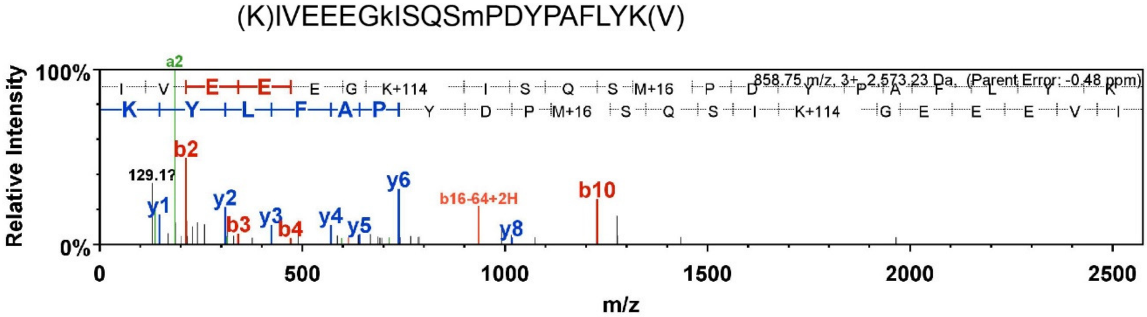

D

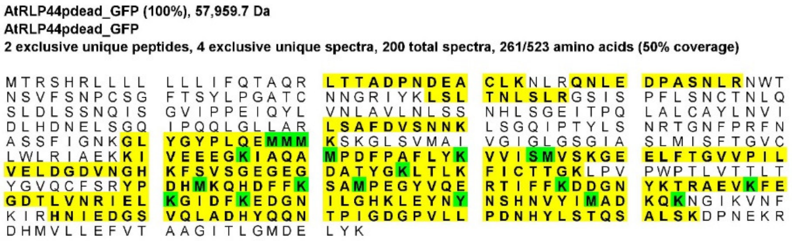

E

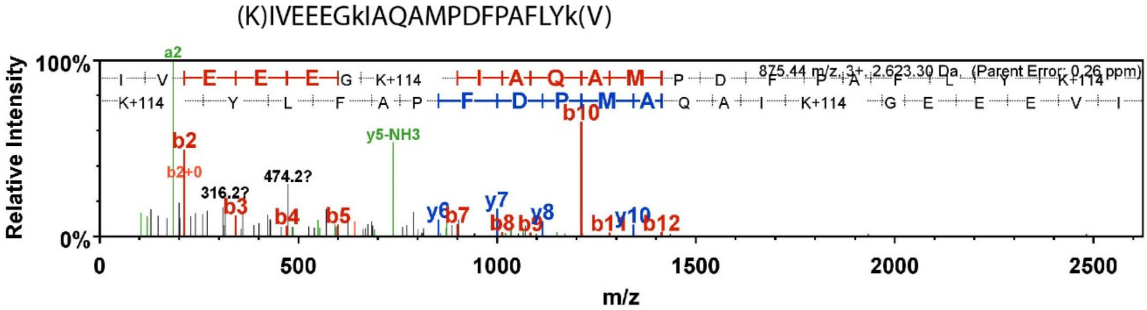

Journal of Cell Science • Supplementary information

Fig. S2. Proteomics analysis of RLP44-GFP during transient expression in *N. benthamiana* and trypsin digestion. Peptide coverage (yellow) and modified amino acids (green) are indicated. Lower panes show spectra of peptides with phosphorylation of S268 and ubiquitination as indicated by double glycine remnant of K266. **(A)** RLP44-GFP WT peptide coverage. **(B)** RLP44-GFP WT peptide with phosphorylation of S268. **(C)** RLP44-GFP WT peptide with ubiquitination of K266. **(D)** RLP44-GFP Pdead peptide coverage. **(E)** RLP44-GFP Pdead peptide with ubiquitination of K266.

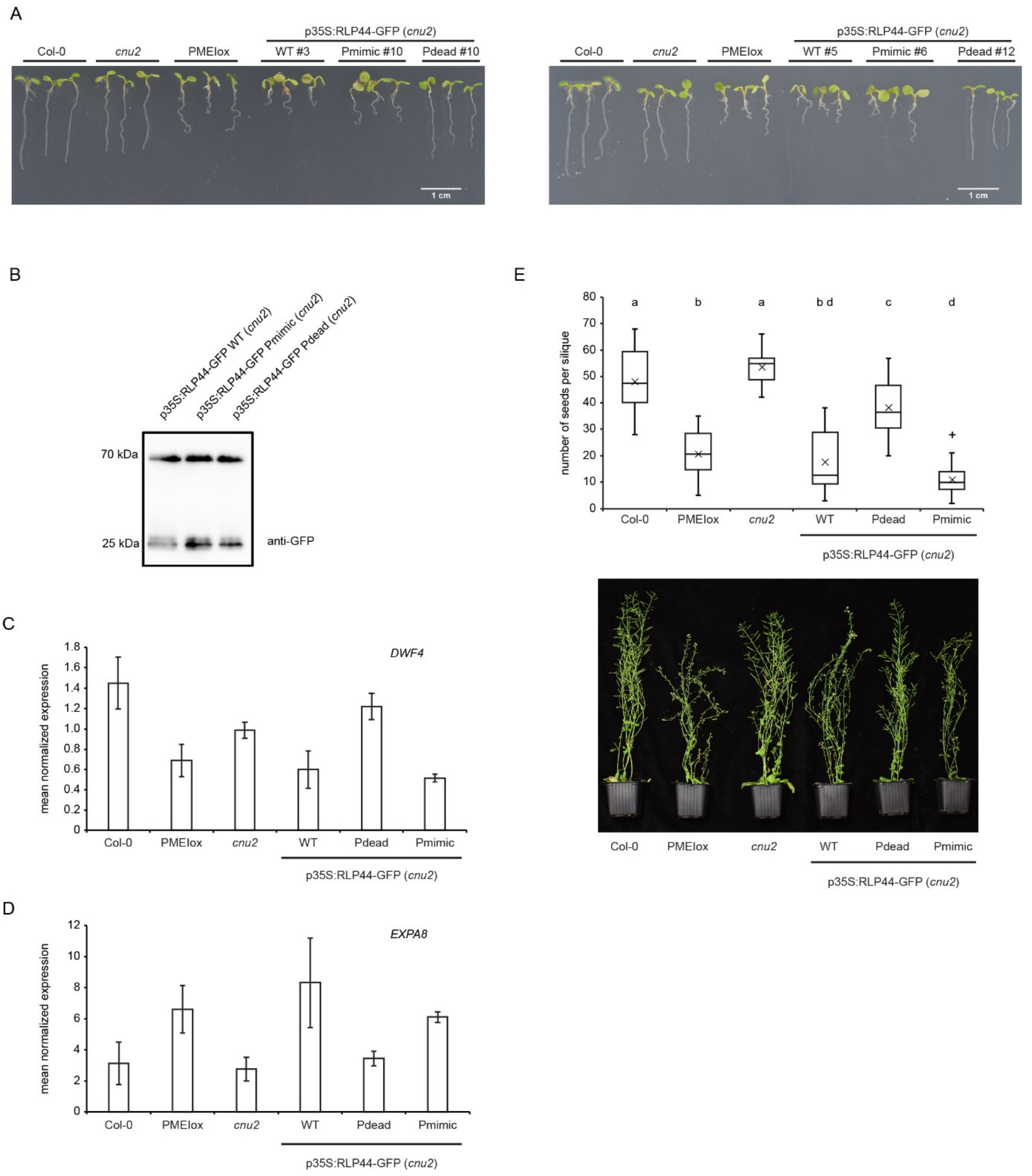

Journal of Cell Science • Supplementary information

Fig. S3. Complementation of *cnu2* by RLP44-GFP WT and RLP44-GFP Pmimic, but not by RLP44-GFP Pdead. (A) Expression of RLP44-GFP WT and Pmimic, but not of Pdead, is able to complement the PMEIox suppressor mutant *cnu2* and leads to recovery of the PMEIox root waving phenotype in seedlings and contorted leaf arrangement in adult plants (see also Fig. 1). **(B)** Lines with comparable RLP44-GFP expression levels were selected based on Western Blot analysis with anti-GFP antiserum. **(C)** Quantitative Real-Time PCR analysis of the BR signalling marker gene *DWF4,* n=3. **(D)** Quantitative Real-Time PCR analysis of the BR signalling marker gene *EXPANSIN8*, n=3. **(E)** Analysis of seed yield in Col-0, PMEIox, *cnu2*, and *cnu2* complementation lines. Boxes indicate range from 25th to 75th percentile, horizontal line indicates the median, whiskers indicate data points within 1.5 times the interquartile range. Markers above whiskers indicate outliers, n = 18. Lettering indicates statistically significant difference according to Tukey's post-hoc test following one-way ANOVA.

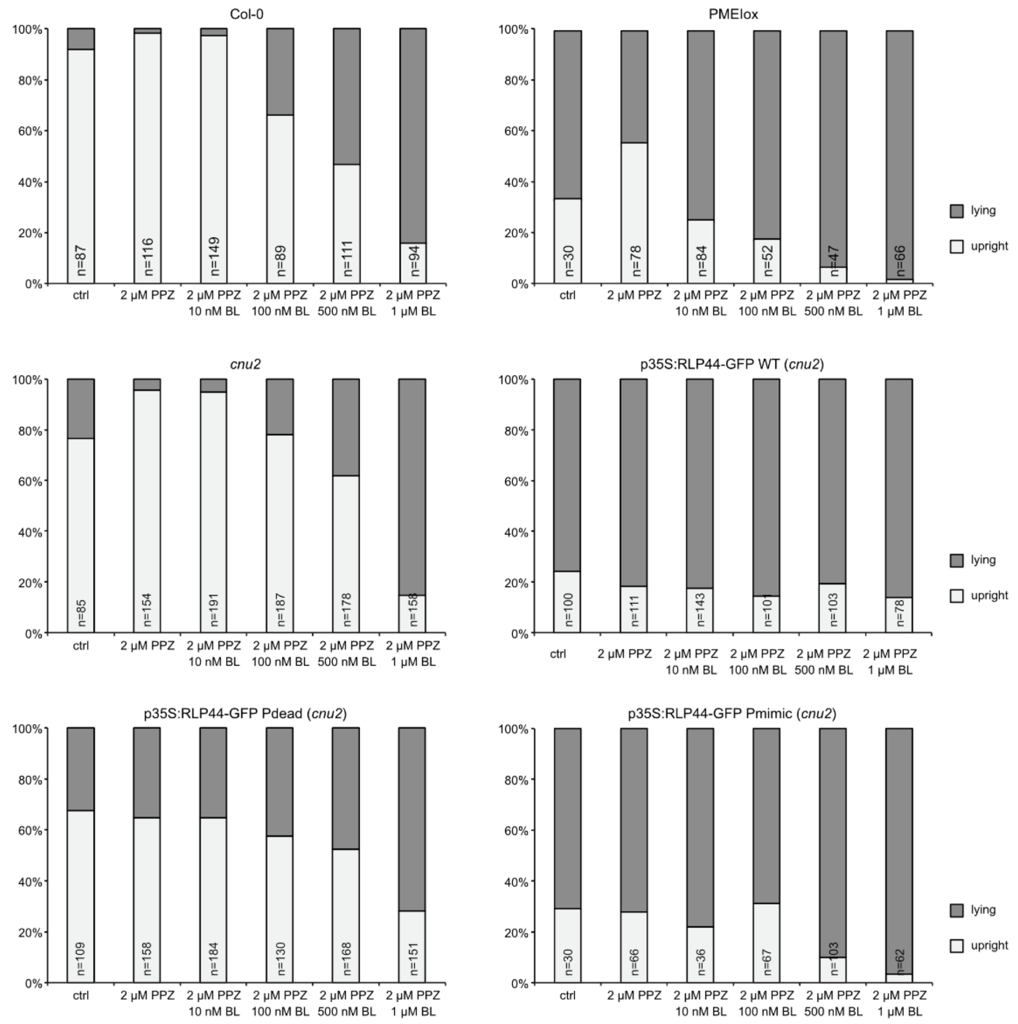

Fig. S4. RLP44-GFP WT and Pmimic, but not RLP44-GFP Pdead restore agravitropic growth of *cnu2* along the surface of agar plates in the dark as observed in PMElox. Endogenous brassinosteroids were depleted with PPZ treatment to test sensitivity to increasing amounts of epi-brassinolide (BL)

Journal of Cell Science • Supplementary information

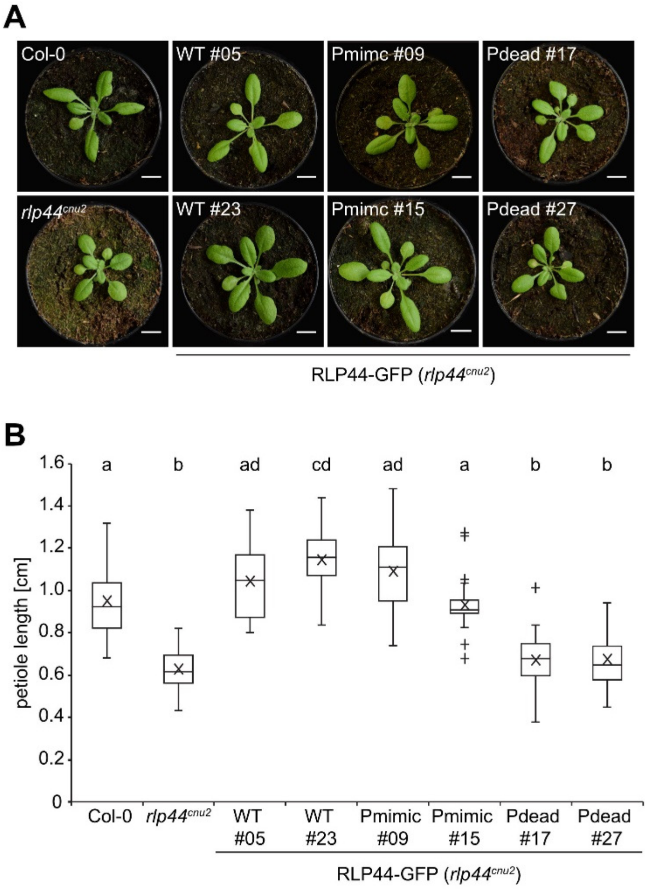

Fig. S5. RLP44-GFP Pdead is unable to complement the petiole phenotype of *rlp44^{cnu2}*. **(A)** Rosette phenotype of Col-0, *rlp44^{cnu2}*, and *rlp44^{cnu2}* complementation lines in long day conditions. **(B)** Quantification of petiole length in lines depicted in A). Boxes indicate range from 25th to 75th percentile, horizontal line indicates the median, whiskers indicate data points within 1.5 times the interquartile range. Markers above whiskers indicate outliers, n = 21. Lettering indicates statistically significant difference according to Tukey's post-hoc test following one-way ANOVA.

Journal of Cell Science • Supplementary information

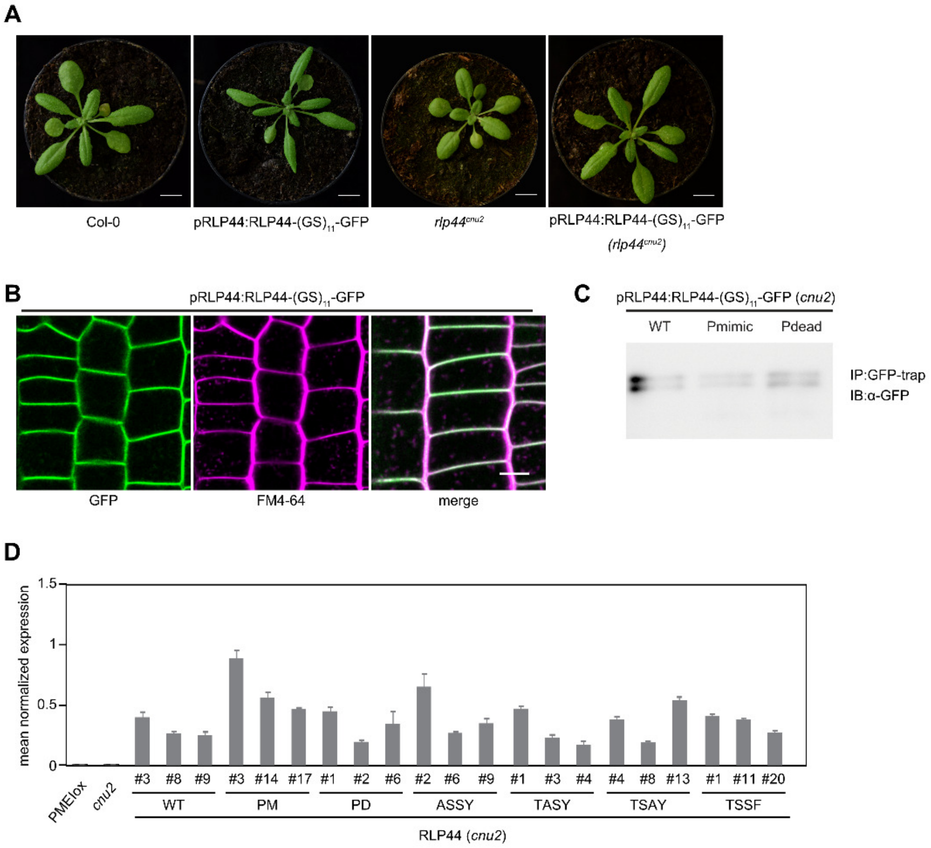

Fig. S6. Overall charge, rather than specific phosphosites modulate RLP44 function. (A) RLP44 overexpression phenotype (see Wolf et al., 2014) of hyperphosphorylated RLP44 under control of its own promoter. **(B)** Plasma membrane localization of hyperphosphorylated pRLP44:RLP44-$(GS)_{11}$-GFP. **(C)** RLP44 Pdead and Pmimic mutants with serine-rich linker show slower migrating band similar to the WT version, suggesting linker phosphorylation. **(D)** Expression of *RLP44* in transgenic lines evaluated in Figure 10 using qPCR (n=3 for each line).

Journal of Cell Science • Supplementary information

Table S1. Mutants and transgenic lines used in this study.

Mutant/transgenic line	
rlp44^{cnu2}	Wolf *et al.*, 2014
cnu2	Wolf *et al.*, 2014
bri1-null	Jaillais *et al.*, 2011a
pskr1-3 pskr2-1	Kutschmar *et al.*, 2009
p35S:RLP44-GFP WT (cnu2)	This study
p35S:RLP44-GFP Pdead (cnu2)	This study
p35S:RLP44-GFP Pmimic (cnu2)	This study
p35S:RLP44-GFP WT	This study
p35S:RLP44-GFP Pdead	This study
p35S:RLP44-GFP Pmimic	This study
p35S:RLP44-GFP WT (*rlp44^{cnu2}*)	This study
p35S:RLP44-GFP Pdead (*rlp44^{cnu2}*)	This study
p35S:RLP44-GFP Pmimic (*rlp44^{cnu2}*)	This study
p35S:RLP44-GFP WT (bri1-null)	Holzwart *et al.*, 2018
p35S:RLP44-GFP Pdead (bri1-null)	This study
p35S:RLP44-GFP Pmimic (bri1-null)	This study
p35S:RLP44-GFP WT (pskr1-3 pskr2-1)	This study
p35S:RLP44-GFP Pdead (pskr1-3 pskr2-1)	This study
pESTR:amiT-TML	Gadeyne *et al.*, 2014
p35S:RLP44-GFP WT (pESTR:amiT-TML)	This study
p35S:RLP44-GFP Pdead (pESTR:amiT-TML)	This study
pRLP44:RLP44 WT (*cnu2*)	This study
pRLP44:RLP44 Pdead (*cnu2*)	This study
pRLP44:RLP44 Pmimic (*cnu2*)	This study
pRLP44:RLP44 T256A (cnu2)	This study
pRLP44:RLP44 S268A (cnu2)	This study
pRLP44:RLP44 S270A (cnu2)	This study
pRLP44:RLP44 Y274F (cnu2)	This study
pRLP44:RLP44-(GS)11-GFP	Holzwart et al., 2018
pRLP44:RLP44-(GS)11-GFP WT (cnu2)	This study
pRLP44:RLP44-(GS)11-GFP Pmimic (cnu2)	This study
pRLP44:RLP44-(GS)11-GFP (Pdead (cnu2)	This study

Journal of Cell Science • Supplementary information

Table S2. Oligonucleotides used in this study

Primer No.	*Primer name*	*Sequence (5' → 3')*	*target*
SW660	RLP44_GW_L	GGGGACAAGTTTGTACAAAAAAGCAGGCTATGA CAAGGAGTCACCGGTTAC	At3g49750
SW670	RLP_GW_WToY _R	GGGGACCACTTTGTACAAGAAAGCTGGGTtGTAA TCAGGCATAGATTGAC	At3g49750
SW666	RLP44_SDMT25 6A_F	gtttatggttgaggattgctgagaagaagattgttg	At3g49750
SW667	RLP44_SDMT25 6A_R	caacaatcttcttctcagcaatcctcaaccataaac	At3g49750
SW668	RLP_S268,270A _Y274F_Rneu	GGGGACCACTTTGTACAAGAAAGCTGGGTagaaa tcaggcatagcttgagcaatcttaccttcttcttcaac	At3g49750
SW672	RLP44_SDMT25 6E_F	GAGGATTGAAGAGAAGAAGATTGTTGAAGAAG	At3g49750
SW673	RLP44_SDMT25 6E_R	ATCTTCTTCTCTTCAATCCTCAACCATAAAC	At3g49750
SW671	RLP_SSY- EEE_R	GGGGACCACTTTGTACAAGAAAGCTGGGTagaaa tcaggcatagattgactaatc	At3g49750
SW661	RLP44_Y274F_ R	GGGGACCACTTTGTACAAGAAAGCTGGGTtttcatc aggcatttcttgttcaatcttaccttcttcttcaac	At3g49750
SW662	RLP44_S268A_ R	GGGGACCACTTTGTACAAGAAAGCTGGGTagtaat caggcatagcttgactaatcttaccttcttc	At3g49750
SW663	RLP44_S270A_ R	GGGGACCACTTTGTACAAGAAAGCTGGGTagtaat caggcatagattgagcaatcttaccttcttcttcaac	At3g49750
SW2446	RLP44-if-f	tgAagcttGGTCTCaGGCTcAATGACAAGGAGTCAC CGG	At3g49750
SW2447	RLP44WT-if-r	atGGCACCCGCCCCTGCTCcGTAATCAGGCATAG ATTG	At3g49750
SW2448	GAGAGA-GFP- if-f	gGAGCAGGGGCGGGTGCC	GFP
SW2449	GAGAGA-GFP- if-r	cgaGAATTcGGTCTCaCTGAttacttgtacagctcgtcc	GFP
SW2450	RLP44TS-AY -if- r	atGGCACCCGCCCCTGCTCcGTAATCAGGCATAG cTTG	At3g49750
SW2452	RLP44TS-SF-if-r	atGGCACCCGCCCCTGCTCcGaAATCAGGCATAG ATTG	At3g49750
SW2454	RLP44AA-AF-if-r	atGGCACCCGCCCCTGCTCcGaAATCAGGCATAG cTTG	At3g49750
SW2455	RLP44EE-EE-if-r	atGGCACCCGCCCCTGCTCcttcATCAGGCATttcTT G	At3g49750
SW3000	RLP44prom_F	GGGGACAAGTTTGTACAAAAAAGCAGGCTTTTG CGATATTTTTGGCTGTC	At3g49750
SW3001	RLP44stop_R	GGGGACCACTTTGTACAAGAAAGCTGGGTTTTA GTAATCAGGCATAGATTGACT	At3g49750
SW3002	RLP44_TASY_st op_R	GGGGACCACTTTGTACAAGAAAGCTGGGTTTTA GTAATCAGGCATAGATTG	At3g49750
SW3003	RLP44_PM_stop _R	GGGGACCACTTTGTACAAGAAAGCTGGGTTTTA TTCATCAGGCATTTCTTGTTCAATCTT	At3g49750
SW3004	RLP44_PD_stop _R	GGGGACCACTTTGTACAAGAAAGCTGGGTTTTA GAAATCAGGCATAGCTTGAGCAATCTT	At3g49750
SW3005	RLP44_TSAY_st op_R	GGGGACCACTTTGTACAAGAAAGCTGGGTTTTA GTAATCAGGCATAGCTTGACTAATCTT	At3g49750
SW3006	RLP44_TSSF_st op_R	GGGGACCACTTTGTACAAGAAAGCTGGGTTTTA GAAATCAGGCATAGATTGACTAATC	At3g49750
SW1179	RLP44_GG_F	AACAGGTCTCAGGCTCAATGACAAGGAGTCACC GGTTA	At3g49750
SW1205	RLP44_GG_R	AACAGGTCTCACTGAGTAATCAGGCATAGATTG AC	At3g49750
SW1367	RLP44_PD_GG C_R	AACAGGTCTCACTGAGAAATCAGGCATAGCTTG	At3g49750

Journal of Cell Science • Supplementary information

SW1368	RLP44_PM_GGC_R	AACAGGTCTCACTGATTCATCAGGCATTTCTTG	At3g49750
SW503	RLP44-4_CAPS_F	AATCTACAAACTCTCACTCAC	At3g49750
SW504	RLP44-4_CAPS_R	CTGACCGGATAATTCGTTATC	At3g49750
SW1377	GK-O8409	ATATTGACCATCATACTCATTGC	
SW1378	GK-134E10_F	TAGCGGAAACAAAATCAGTGG	At4g39400
SW1379	GK-134E10_R	TCGTTCCATTGAAGAGATTGG	At4g39400
SW1754	pskr1-3_F	CTCGCTTTCTGGTATGACGAG	At2g02220
SW1746	pskr1-3_R	TCCGAAACTATACACATCGCC	At2g02220
SW1984	pskr2-1_F	TTCTTAGACTGTTTGGCTCGG	At5g53890
SW1985	pskr2-1_R	GCGTTACAAACATGCAACAAG	At5g53890
SW230	LBb1.3	ATTTTGCCGATTTCGGAAC	
SW905	attB1	ACAAGTTTGTACAAAAAAGCAGGCT	
SW906	attB2	ACCACTTTGTACAAGAAAGCTGGGT	
SW1202	pGG-Bdummy_F	GTATTCAGTCGACTGGTACCAAC	
SW1137	pGGA/C000_R	CAGATTGTACTGAGAGTGCACC	
SW521	AtEXP8_F	CCGAAATAACTAACCCCTCCTC	At2g40610
SW522	AtEXP8_R	TAGCCACAAGCTCCGCCCAT	At2g40610
SW803	DWF4_F	CAACAGCAAAACAACGGAGCG	At3g50660
SW804	DWF4_R	TCTGAACCAGCACATAGCCTTG	At3g50660
SW1015	Clath_F	TCGATTGCTTGGTTTGGAAGAT	At1g10730
SW1016	Clath_R	GCACTTAGCGTGGACTCTGTTTGC	At1g10730
SW612	RLP44COD2_F	TCAGATTCCGCAGCAATTAG	At3g49750
SW613	RLP44COD2_R	TCCTGCAACGGATAACCATA	At3g49750
	ACT2_F	CAGTGTCTGGATCGGTGGTT	At3g18780
	ACT2_R	TGAACGATTCCTGGACCTGC	At3g18780

Journal of Cell Science • Supplementary information

Table S3. Overview of constructs generated with GreenGate cloning.

**pSW362**	**pRLP44:RLP44-$(GS)_{11}$-GFP WT**			
	Name	*Internal name*	*Source*	*Primers*
"Promoter" module	pRLP44	pSW299	Holzwart et al., 2018	
"N-tag" module	B-dummy	pGGB003	Lampropoulos et al., 2013	
"CDS" module	RLP44	pSW334	Holzwart et al., 2018	SW1179-1205
"C-tag" module	$(GS)_{11}$-GFP	PGGD001	Lampropoulos et al., 2013	
"Terminator" module	tUBQ10	pGGE009	Lampropoulos et al., 2013	
"Resistance" module	SulfR	pGGF006	Lampropoulos et al.,2013	
Destination vector		pGGZ0001	Lampropoulos et al.,2013	
**pSW567**	**pRLP44:RLP44-$(GS)_{11}$-GFP Pmimic**			
	Name	*Internal name*	*Source*	*Primers*
"Promoter" module	pRLP44	pSW299	Holzwart et al., 2018	
"N-tag" module	B-dummy	pGGB003	Lampropoulos et al., 2013	
"CDS" module	RLP44pmimic	pSW519	This study	SW1179-1368
"C-tag" module	$(GS)_{11}$-GFP	PGGD001	Lampropoulos et al., 2013	
"Terminator" module	tUBQ10	pGGE009	Lampropoulos et al., 2013	
"Resistance" module	SulfR	pGGF006	Lampropoulos et al.,2013	
Destination vector		pGGZ0001	Lampropoulos et al.,2013	
**pSW566**	**pRLP44:RLP44-$(GS)_{11}$-GFP Pdead**			
	Name	*Internal name*	*Source*	*Primers*
"Promoter" module	pRLP44	pSW299	Holzwart et al., 2018	
"N-tag" module	B-dummy	pGGB003	Lampropoulos et al., 2013	
"CDS" module	RLP44pdead	pSW518	This study	SW1179-1367
"C-tag" module	$(GS)_{11}$-GFP	PGGD001	Lampropoulos et al., 2013	
"Terminator" module	tUBQ10	pGGE009	Lampropoulos et al., 2013	
"Resistance" module	SulfR	pGGF006	Lampropoulos et al.,2013	
Destination vector		pGGZ0001	Lampropoulos et al.,2013	
**pSW1027**	**pRLP44:RLP44-GAGAGA-GFP WT**			
"Promoter" module	pRLP44	pSW299	Holzwart et al., 2018	
"N-tag" module	B-dummy	pGGB003	Lampropoulos et al., 2013	
"CDS" module	RLP44-GAGAGA-GFP	pSW999	This study	SW2446-2449
"C-tag" module	D-dummy	pGGD002	Lampropoulos et al., 2013	
"Terminator" module	tUBQ10	pGGE009	Lampropoulos et al., 2013	
"Resistance" module	SulfR	pGGF006	Lampropoulos et al.,2013	
Destination vector		pGGZ0001	Lampropoulos et al.,2013	
**pSW1019**	**pRLP44:RLP44-GAGAGA-GFP Pmimic**			
"Promoter" module	pRLP44	pSW299	Holzwart et al., 2018	
"N-tag" module	B-dummy	pGGB003	Lampropoulos et al., 2013	
"CDS" module	RLP44-GAGAGA-GFP Pmimic	pSW1018	This study	SW2446-2455, 2448+2449
"C-tag" module	D-dummy	pGGD002	Lampropoulos et al., 2013	
"Terminator" module	tUBQ10	pGGE009	Lampropoulos et al., 2013	
"Resistance" module	SulfR	pGGF006	Lampropoulos et al.,2013	
Destination vector		pGGZ0001	Lampropoulos et al.,2013	
**pSW1017**	**pRLP44:RLP44-GAGAGA-GFP Pdead**			
"Promoter" module	pRLP44	pSW299	Holzwart et al., 2018	
"N-tag" module	B-dummy	pGGB003	Lampropoulos et al., 2013	

Journal of Cell Science • Supplementary information

“CDS” module	RLP44-GAGAGA-GFP Pdead	pSW1007	This study	SW2446+2454, 2448+2449
“C-tag” module	D-dummy	pGGD002	Lampropoulos et al., 2013	
“Terminator” module	tUBQ10	pGGE009	Lampropoulos et al., 2013	
“Resistance” module	SulfR	pGGF006	Lampropoulos et al.,2013	
Destination vector		pGGZ0001	Lampropoulos et al.,2013	
**pSW1026**	**pRLP44:RLP44-GAGAGA-GFP ASSY**			
“Promoter” module	pRLP44	pSW299	Holzwart et al., 2018	
“N-tag” module	B-dummy	pGGB003	Lampropoulos et al., 2013	
“CDS” module	RLP44-GAGAGA-GFP ASSY	pSW1000	This study	SW2446+2447, 2448+2449
“C-tag” module	D-dummy	pGGD002	Lampropoulos et al., 2013	
“Terminator” module	tUBQ10	pGGE009	Lampropoulos et al., 2013	
“Resistance” module	SulfR	pGGF006	Lampropoulos et al.,2013	
Destination vector		pGGZ0001	Lampropoulos et al.,2013	
**pSW1028**	**pRLP44:RLP44-GAGAGA-GFP TASY**			
“Promoter” module	pRLP44	pSW299	Holzwart et al., 2018	
“N-tag” module	B-dummy	pGGB003	Lampropoulos et al., 2013	
“CDS” module	RLP44-GAGAGA-GFP TASY	pSW1002	This study	SW2446+2447, 2448+2449
“C-tag” module	D-dummy	pGGD002	Lampropoulos et al., 2013	
“Terminator” module	tUBQ10	pGGE009	Lampropoulos et al., 2013	
“Resistance” module	SulfR	pGGF006	Lampropoulos et al.,2013	
Destination vector		pGGZ0001	Lampropoulos et al.,2013	
**pSW1014**	**pRLP44:RLP44-GAGAGA-GFP TSAY**			
“Promoter” module	pRLP44	pSW299	Holzwart et al., 2018	
“N-tag” module	B-dummy	pGGB003	Lampropoulos et al., 2013	
“CDS” module	RLP44-GAGAGA-GFP TSAY	pSW1004	This study	SW2446+2450, 2448+2449
“C-tag” module	D-dummy	pGGD002	Lampropoulos et al., 2013	
“Terminator” module	tUBQ10	pGGE009	Lampropoulos et al., 2013	
“Resistance” module	SulfR	pGGF006	Lampropoulos et al.,2013	
Destination vector		pGGZ0001	Lampropoulos et al.,2013	
**pSW1015**	**pRLP44:RLP44-GAGAGA-GFP TSSF**			
“Promoter” module	pRLP44	pSW299	Holzwart et al., 2018	
“N-tag” module	B-dummy	pGGB003	Lampropoulos et al., 2013	
“CDS” module	RLP44-GAGAGA-GFP TSSF	pSW1005	This study	SW2446+2452, 2448+2449
“C-tag” module	D-dummy	pGGD002	Lampropoulos et al., 2013	
“Terminator” module	tUBQ10	pGGE009	Lampropoulos et al., 2013	
“Resistance” module	SulfR	pGGF006	Lampropoulos et al.,2013	
Destination vector		pGGZ0001	Lampropoulos et al.,2013	

Journal of Cell Science • Supplementary information
