# Supplementary material for: Phosphorylation-dependent routing of RLP44 towards brassinosteroid or phytosulfokine signalling
Source: J Cell Sci. 2021 Oct 20;134(20):jcs259134. doi: 10.1242/jcs.259134 (PMC8572011; doi:10.1242/jcs.259134)
Supplement: Reviewer comments [file joces-134-259134_review_history.pdf]

Phosphorylation-dependent routing of RLP44 towards brassinosteroid or phytosulfokine signalling

Borja Garnelo Gómez, Eleonore Holzwart, Chaonan Shi, Rosa Lozano-Durán and Sebastian Wolf
DOI: 10.1242/jcs.259134

Editor: Mahak Sharma

Review timeline
Original submission: 13 July 2021
Editorial decision: 31 August 2021
First revision received: 2 September 2021
Accepted: 20 September 2021

Original submission

First decision letter

MS ID#: JOCES/2021/259134

MS TITLE: Phosphorylation-dependent routing of RLP44 towards brassinosteroid or phytosulfokine signalling

AUTHORS: Eleonore Holzwart, Borja Garnelo Gomez, Chaonan Shi, Rosa Lozano-Duran, and Sebastian Wolf
ARTICLE TYPE: Research Article

We have now reached a decision on the above manuscript.

To see the reviewers' reports and a copy of this decision letter, please go to: https://submit-jcs.biologists.org and click on the 'Manuscripts with Decisions' queue in the Author Area. (Corresponding author only has access to reviews.)

As you will see, the reviewers found the study to be quite interesting and they also appreciated that experiments were well executed and support the major conclusions of your study. From the reviewers' comments, you will see that there are few important points including distribution of RLP44 in BR1 null cells and phosphorylation status of different RLP44 alleles that require amendments to your manuscript. I hope that you will be able to carry these out, because I would like to be able to accept your paper.

We are aware that you may be experiencing disruption to the normal running of your lab that makes experimental revisions challenging. If it would be helpful, we encourage you to contact us to discuss your revision in greater detail. Please send us a point-by-point response indicating where you are able to address concerns raised (either experimentally or by changes to the text) and where you will not be able to do so within the normal timeframe of a revision. We will then provide further guidance. Please also note that we are happy to extend revision timeframes as necessary.

Please ensure that you clearly highlight all changes made in the revised manuscript. Please avoid using 'Tracked changes' in Word files as these are lost in PDF conversion.

© 2021. Published by The Company of Biologists under the terms of the Creative Commons Attribution License (https://creativecommons.org/licenses/by/4.0/).

I should be grateful if you would also provide a point-by-point response detailing how you have dealt with the points raised by the reviewers in the 'Response to Reviewers' box. Please attend to all of the reviewers' comments. If you do not agree with any of their criticisms or suggestions please explain clearly why this is so.

Reviewer 1

Advance summary and potential significance to field

The manuscript by Holzwart et al investigates how RLP44 can function in two distinct signaling pathways, namely phytosulfokine and Brassinostroids. They identify this may have to do with distinct subcellular localisation that is dependent on charge generated by phosphorylation with increased phosphorylation resulting in preferential PM localisation. This is a very interesting observation and provides insights into how same components can function in distinct pathways.

Comments for the author

1. “Here, we show that RLP44 is phosphorylated in its highly conserved C-terminal cytosolic tail. This post-translational modification is crucial for regulating RLP44’s function in BR signalling activation. RLP44 variants in which phosphorylation is blocked enter endocytosis prematurely, leading to an almost entirely intracellular localization. Conversely, mimicking phosphorylation or ectopic phosphorylation results in preferential RLP44 localization at the plasma membrane. This increase in the ratio of plasma membrane to intracellular localization is controlled by phospho-charge, rather than by modification of specific amino acids and is furthermore dependent on the presence of BRI1, suggesting that phosphorylation affects subcellular localization through modulating the interactions of the LRR proteins. In contrast, RLP44’s role in PSK signalling is not affected by phospho-status. Thus, our results provide a framework to understand how specificity can be determined in plasma membrane receptor complex interactions.”

   This is repetitive with abstract and can be shortened. as can be the rest of introduction.

2. I agree that the data suggests that increased endosomal trafficking might explain RLPdead phenotype but can the authors really exclude reduced secretion of this variant? I mean that blocking endocytosis would generally increase PM levels of most proteins that are endocytosed right?

3. This is very interesting data. However I wonder why the wild type RLP44 levels dont decrease in bri1 mutant background? Can the aurthors comment on steady state levels of RLP44 phosphorylation?

Reviewer 2

Advance summary and potential significance to field

The manuscript described the localization and function of RLP44 dependent on the phosphorylation. By using the PMEIox cnu2 line, RLP44 Pmimic but not Pdead version was shown to be functional in BR signaling.

Analysis of RLP44-GFP localization showed intracellular localization of the Pdead version and exclusive PM localization of the Pmimic version. Then, the analysis using wormannin, BFA, and TML amiRNA showed that the Pdead version is efficiently internalized by endocytosis from the PM. In addition, the Pmimic version showed enhanced internalization in the bri1-null mutant. This result suggested that presence of BRI1 inhibits the endocytosis of the phosphorylated RLP44. The analysis of the phenotypes dependent on the PSK signaling showed that Pmimic and Pdead did not largely affect the function of RLP44. This result suggested that the phosphorylation is not required for the role of RLP44 in PSK signaling. The experiments using the Pdead RLP44-GFP with GS11 linker showed that phosphorylation in the linker resulted in the PM localization and the functional

© 2021. Published by The Company of Biologists under the terms of the Creative Commons Attribution License (https://creativecommons.org/licenses/by/4.0/).

complementation of the cnu2 mutant. This result suggested that the phosphor-charge is responsible for the RLP44 function in BR signaling. Finally the transgenics expressing untagged RLP44 constructs confirmed importance of the phosphosites on the RLP44 function in the BR signaling. Although interaction of RLP44 with BRI1 and PSKR1 was not directly examined in this manuscript, the conclusion availability of RLP4 to engage with the BRI1 or PSKR1 receptor complexes is differently modulated by phosphorylation, is well supported by the results. I agree that this system will be an attractive model of phosphorylation-dependent changes of PM receptor complex interactions.

Comments for the author

Minor comments
- Page 5 “rlp44 mutant”: Please describe the type of mutant (loss-of function? Point mutation?).
- Fig.2C: Please describe the method to measure PM/intracellular signal ratio.

<u>Reviewer 3</u>

Advance summary and potential significance to field

Signaling via LRR-RLKs involves highly complex, multifaceted crosstalk that appears tightly controlled in a spatiotemporal context to establish specificity in such signaling. Elucidation of mechanisms that define such specificity, however, remains a challenge.

In this manuscript, the authors address regulation of signaling adaptor RLP44, and its functions, both in brassinolide as well as phytosulfokine signal transduction. IP-MS revealed evidence for protein phosphorylation which led the authors to analyzing four putative phosphorylation sites, by employing either phospho-dead or phosphomimic rlp44 mutant alleles. Only phosphomimic rlp44 retained functionality in BR signaling, whilst the phospho-dead version failed to rescue the rlp44cnu2 allele. The authors then convincingly demonstrated that phosphor-dead rlp44 exhibits endosomal rather than plasma membrane-associated subcellular localization. Pharmacological and genetic analyses is provided, suggesting that such mislocalization is due to enhanced endocytic sorting, via clathrin-mediated endocytosis, which presumably affects RLP44-BRI1 crosstalk at the plasma membrane.

importantly, phytosulfokine-induced signaling appears unaffected by the mutations introduced, indicative of RLP44 phosphorylation acting specifically in mediating brassinolide signals. The authors went one step further, analyzing RLP44-GFP reporters with a serin-rich linker introduced between RLP44 and the reporter protein. This linker appears hyperphosphorylated, which, according to the authors’ experiments would be sufficient to maintain functionality in brassinolide signaling. Further experiments, with untagged RLP44 versions being mutated at relevant phosphorylation sites, further supported a crucial role for protein phosphorylation in the control of RLP44 function. Together, all these observations led the authors to the conclusion that it is the overall phosphorylation status, rather than phosphorylation at specific sites, that controls localization and hence functionality of RLP44 in brassinolide signaling.

Comments for the author

Overall, I am quite happy with this manuscript, as it represents a valuable contribution to our understanding of specificity in RLK/RLP signaling.
Two things, however, appear a bit vague and might require some additional input from the authors.

i) on page 8, the authors analyze the role of BRI1 in the control of RLP44. Here, the authors found that localization of wild type RLP44:GFP and rlp44-phospho-dead:GFP is not affected in a bri1 null allele.
Distribution of rlp4-phosphomimic:GFP signals, however, is shifted from the plasma membrane to the cells’ interior. This is an interesting observation, and the authors concluded that BRI1 protein in one way or another modulates endocytic sorting of phosphorylated RLP44. On the other hand, distribution of wild type RLP44:GFP, which is functional in brassinolide signaling (as is rlp44-

© 2021. Published by The Company of Biologists under the terms of the Creative Commons Attribution License (https://creativecommons.org/licenses/by/4.0/).

phosphomimic:GFP) does not respond to the loss of BRI1. How would the authors explain this discrepancy?

ii) on page 9 and 10, the authors describe their analysis of RLP44-(GS)-GFP which has a serine-rich linker positioned between RLP44 and GFP. The authors provide strong biochemical evidence that this linker is phosphorylated in planta. In addition, the authors tested functionality of additional RLP44-(GS)-GFP fusion proteins, in which the serin linker has been introduced into their original phospho-dead and phosphomimic rlp44 alleles. Expression of these alleles, which locate preferentially to the plasma membrane rescues cnu2 phenotypes, which is indeed strong evidence for overall RLP44 phosphorylation (charge?) acting as effector of subcellular localization and functionality in brassinolide signaling. Maybe I missed that, but did the authors test the phosphorylation status of these alleles – similar to the phosphatase treatment provided for the wild type RLP44-(GS)-GFP fusion protein?

First revision

Author response to reviewers' comments

We would like to thank all reviewers for their constructive and helpful comments. Here is our point-by-point reponse to all issues raised.

Reviewer 1 (extracted from uploaded file)
1. This [marked text at end of introduction] is repetitive with abstract and can be shortened. as can be the rest of introduction.

>> We considerably shortened the passage in question to avoid duplications and performed minor edits to reduce the length of the introduction. We also shortened the abstract to comply with the 180 word limit.

2. I agree that the data suggests that increased endosomal trafficking might explain RLPdead phenotype but can the authors really exclude reduced secretion of this variant? I mean that blocking endocytosis would generally increase PM levels of most proteins that are endocytosed right?

>> We agree with the reviewer that blocking endocytosis would increase PM-to-cytosol ratios of most proteins, as can be seen for the RLP44 WT protein. Therefore, we cannot formally exclude reduced secretion of the RLP44 Pdead variant, even though the BFA experiments also point towards enhanced endocytosis. We have amended the text, which is now more carefully phrased and mentions this caveat.

3. This is very interesting data. However I wonder why the wild type RLP44 levels dont decrease in bri1 mutant background? Can the aurthors comment on steady state levels of RLP44 phosphorylation?

>> At present, we cannot completely explain the absence of a large effect on RLP44 WT levels at the PM by BRI1. However, we felt that it was important to include those data to underline the complexity of the interplay of protein interactions and regulation of trafficking, even though we cannot provide mechanistic insight at this point. We have rephrased the respective section of the manuscript to draw the reader's attention to the (lack of) effect on the RLP44 WT fusion protein and mention that interpretations in the bri-null could be impeded by the wide-ranging transcriptional rearrangements in this background and potential direct and indirect effects on the composition of LRR-RLK complexes by the absence of BRI1. The same caveats apply to results we have obtained with Western Blotting that suggest that the pRLP44-(GS)-GFP fusion protein is still phosphorylated in the bri1-null background, which is why they are not included here.

Reviewer 2 Advance summary and potential significance to field

© 2021. Published by The Company of Biologists under the terms of the Creative Commons Attribution License (https://creativecommons.org/licenses/by/4.0/).

The manuscript described the localization and function of RLP44 dependent on the phosphorylation. By using the PMEIox cnu2 line, RLP44 Pmimic but not Pdead version was shown to be functional in BR signaling.

Analysis of RLP44-GFP localization showed intracellular localization of the Pdead version and exclusive PM localization of the Pmimic version. Then, the analysis using wormannin, BFA, and TML amiRNA showed that the Pdead version is efficiently internalized by endocytosis from the PM. In addition, the Pmimic version showed enhanced internalization in the bri1-null mutant. This result suggested that presence of BRI1 inhibits the endocytosis of the phosphorylated RLP44. The analysis of the phenotypes dependent on the PSK signaling showed that Pmimic and Pdead did not largely affect the function of RLP44. This result suggested that the phosphorylation is not required for the role of RLP44 in PSK signaling. The experiments using the Pdead RLP44-GFP with GS11 linker showed that phosphorylation in the linker resulted in the PM localization and the functional complementation of the cnu2 mutant. This result suggested that the phosphor-charge is responsible for the RLP44 function in BR signaling. Finally the transgenics expressing untagged RLP44 constructs confirmed importance of the phosphosites on the RLP44 function in the BR signaling. Although interaction of RLP44 with BRI1 and PSKR1 was not directly examined in this manuscript, the conclusion, availability of RLP4 to engage with the BRI1 or PSKR1 receptor complexes is differently modulated by phosphorylation, is well supported by the results. I agree that this system will be an attractive model of phosphorylation-dependent changes of PM receptor complex interactions.

Reviewer 2 Comments for the author
Minor comments
-Page 5 “rlp44 mutant”: Please describe the type of mutant (loss-of function? Point mutation?).

>> cnu2 and rlp44cnu2 carry a point mutation leading to a premature stop codon. Complementation assays and comparison with the transcriptional null allele rlp44-3 (a T-DNA) insertions suggest that cnu2 and rlp44cnu2 are loss of function mutants (Wolf et al., 2014). We have amended the text accordingly.

-Fig.2C: Please describe the method to measure PM/intracellular signal ratio.

>> We apologize for this omission and now include a full description of the method in the Materials and Methods section.

Reviewer 3 Advance summary and potential significance to field
Signaling via LRR-RLKs involves highly complex, multifaceted crosstalk that appears tightly controlled in a spatiotemporal context to establish specificity in such signaling. Elucidation of mechanisms that define such specificity, however, remains a challenge.

In this manuscript, the authors address regulation of signaling adaptor RLP44, and its functions, both in brassinolide as well as phytosulfokine signal transduction. IP-MS revealed evidence for protein phosphorylation which led the authors to analyzing four putative phosphorylation sites, by employing either phospho-dead or phosphomimic rlp44 mutant alleles. Only phosphomimic rlp44 retained functionality in BR signaling, whilst the phospho-dead version failed to rescue the rlp44cnu2 allele. The authors then convincingly demonstrated that phosphor-dead rlp44 exhibits endosomal rather than plasma membrane-associated subcellular localization. Pharmacological and genetic analyses is provided, suggesting that such mislocalization is due to enhanced endocytic sorting, via clathrin-mediated endocytosis, which presumably affects RLP44-BRI1 crosstalk at the plasma membrane.importantly, phytosulfokine-induced signaling appears unaffected by the mutations introduced, indicative of RLP44 phosphorylation acting specifically in mediating brassinolide signals. The authors went one step further, analyzing RLP44-GFP reporters with a serin-rich linker introduced between RLP44 and the reporter protein. This linker appears hyperphosphorylated, which, according to the authors’ experiments would be sufficient to maintain functionality in brassinolide signaling. Further experiments, with untagged RLP44 versions being mutated at relevant phosphorylation sites, further supported a crucial role for protein phosphorylation in the control of RLP44 function. Together, all these observations led the authors to the conclusion that it is the overall phosphorylation status, rather than phosphorylation at specific sites, that controls localization and hence functionality of RLP44 in brassinolide signaling.
Reviewer 3 Comments for the author

© 2021. Published by The Company of Biologists under the terms of the Creative Commons Attribution License (https://creativecommons.org/licenses/by/4.0/).

Overall, I am quite happy with this manuscript, as it represents a valuable contribution to our understanding of specificity in RLK/RLP signaling.

Two things, however, appear a bit vague and might require some additional input from the authors.
i)on page 8, the authors analyze the role of BRI1 in the control of RLP44. Here, the authors found that localization of wild type RLP44:GFP and rlp44-phospho-dead:GFP is not affected in a bri1 null allele.
Distribution of rlp4-phosphomimic:GFP signals, however, is shifted from the plasma membrane to the cells' interior. This is an interesting observation, and the authors concluded that BRI1 protein in one way or another modulates endocytic sorting of phosphorylated RLP44. On the other hand, distribution of wild type RLP44:GFP, which is functional in brassinolide signaling (as is rlp44-phosphomimic:GFP) does not respond to the loss of BRI1. How would the authors explain this discrepancy?

>> (See also similar comment by reviewer 1) At present, we cannot explain the absence of a large effect on RLP44 WT levels at the PM by BRI1, but felt that it was important to include those data to underline the complexity of the interplay of protein interactions and regulation of trafficking, even though we cannot provide a mechanistic at this point. We have rephrased the respective section of the manuscript to draw the reader's attention to the (lack of) effect on the RLP44 WT fusion protein and mention that interpretations in the bri-null could be impeded by the wide-ranging transcriptional rearrangements in this background and potential direct and indirect effects on the composition of LRR-RLK complexes by the absence of BRI1.

ii)on page 9 and 10, the authors describe their analysis of RLP44-(GS)-GFP which has a serine-rich linker positioned between RLP44 and GFP. The authors provide strong biochemical evidence that this linker is phosphorylated in planta. In addition, the authors tested functionality of additional RLP44-(GS)-GFP fusion proteins, in which the serin linker has been introduced into their original phospho-dead and phosphomimic rlp44 alleles. Expression of these alleles, which locate preferentially to the plasma membrane rescues cnu2 phenotypes, which is indeed strong evidence for overall RLP44 phosphorylation (charge?) acting as effector of subcellular localization and functionality in brassinolide signaling. Maybe I missed that, but did the authors test the phosphorylation status of these alleles – similar to the phosphatase treatment provided for the wild type RLP44-(GS)-GFP fusion protein?

>> We thank the reviewer for this observation and now provide a Western blot result in Figure S6C, demonstrating the presence of a slower migrating form of the RLP44-(GS)-GFP Pdead and Pmimic variants, similar in migrating behaviour to the band demonstrated to be a phosphorylated form of RLP44-(GS)-GFP WT. Thus, these results suggest that both mutant versions are phosphorylated, in agreement with our other results.

Second decision letter

MS ID#: JOCES/2021/259134

MS TITLE: Phosphorylation-dependent routing of RLP44 towards brassinosteroid or phytosulfokine signalling

AUTHORS: Borja Garnelo Gomez, Eleonore Holzwart, Chaonan Shi, Rosa Lozano-Duran, and Sebastian Wolf
ARTICLE TYPE: Research Article

I am happy to tell you that your manuscript has been accepted for publication in Journal of Cell Science, pending standard ethics checks.

Reviewer 2

© 2021. Published by The Company of Biologists under the terms of the Creative Commons Attribution License (https://creativecommons.org/licenses/by/4.0/).

Advance summary and potential significance to field

The paper showed that availability of RLP4 to engage with the BRI1 or PSKR1 receptor complexes is differently modulated by phosphorylation. I agree that this system will be an attractive model of phosphorylation-dependent changes of PM receptor complex interactions.

Comments for the author

I think the authors answered the comments by reviewers satisfactory.

Reviewer 3

Advance summary and potential significance to field

see my comments on the original submission.

Comments for the author

All my minor concerns have been addressed appropriately.

© 2021. Published by The Company of Biologists under the terms of the Creative Commons Attribution License (https://creativecommons.org/licenses/by/4.0/).
